# Associations of Creatinine Muscle Index with markers of sarcopenia and mortality in chronic kidney disease: A prospective cohort study

**DOI:** 10.1371/journal.pmed.1004775

**Published:** 2026-02-12

**Authors:** Thomas McDonnell, Thomas Phillips, Philip A. Kalra, Simon D. S. Fraser, Rosamonde E. Banks, Nicolas Vuilleumier, Maarten W. Taal, on behalf of the NURTuRE-CKD academic steering group

**Affiliations:** 1 Donal O’Donoghue Renal Research Centre, Salford Royal Hospital, Northern Care Alliance NHS Foundation Trust, Salford, United Kingdom; 2 University of Manchester, Faculty of Biology Medicine and Health, University of Manchester, Manchester, United Kingdom; 3 School of Primary Care, Population Sciences and Medical Education, Faculty of Medicine, University of Southampton, Southampton, United Kingdom; 4 Leeds Institute of Medical Research, St James’s University Hospital, School of Medicine, Leeds, United Kingdom; 5 Division of Laboratory Medicine, Diagnostic Department, Geneva University Hospitals and Faculty of Medicine, Geneva, Switzerland; 6 Department of Renal Medicine, University Hospitals of Derby and Burton NHS Foundation Trust, Derby, United Kingdom; 7 Centre for Kidney Research and Innovation, Academic Unit for Translational Medical Sciences, School of Medicine, University of Nottingham, Nottingham, United Kingdom; Istituto Di Ricerche Farmacologiche Mario Negri, ITALY

## Abstract

**Background:**

Sarcopenia is common in chronic kidney disease (CKD) and linked to higher mortality, but identifying those at risk remains challenging. Indices combining serum creatinine and cystatin C (eGFRratio and eGFRdifference) have been studied, but have tended to perform worse in those with CKD. This study aimed to examine the relationship of Creatinine Muscle Index (CMI), an estimate of glomerular filtration of creatinine, with sarcopenia and mortality in a non-dialysis CKD population.

**Methods and findings:**

NURTuRE-CKD is a prospective, multicentre cohort study of people with non-dialysis CKD in the UK. Two thousand nine hundred ninety-six individuals were enrolled between July 2017 and September 2019. Cystatin C measurements were available in 2,930 adults. CMI (mg/day) was calculated as eGFR cystatin C × serum creatinine concentration. The relationships between CMI and: (1) probable sarcopenia (defined as the best hand grip strength of <27 kg for males and <16 kg for females); (2) individual muscle function measures including hand grip strength (kg) and timed get-up-and-go (TUG) (seconds); (3) all-cause mortality were assessed using Spearman’s correlation, logistic regression, and Cox proportional hazards models, stratified by sex and adjusted for age, ethnicity, body mass index, smoking status, Charlson Comorbidity Index, urine albumin-to-creatinine ratio, and C-reactive protein. TUG test is the time taken to stand from a chair, walk 3 m, turn, return, and sit down. Among 1,723 males and 1,207 females, the median (IQR) age was 66 (53–74) years, and the median eGFRcreatinine was 34 (24–47) ml/min/1.73m^2^. A total of 806 participants (27.5%) had probable sarcopenia, and over a median follow-up period of 50 (41 to 56) months, 527 (18%) died. The adjusted OR for probable sarcopenia per 100 mg/day increase in CMI was OR 0.72 (95% CI 0.67, 0.78 *p* value <0.001) in males and OR 0.81 (95% CI 0.73, 0.89 *p* value < 0.001) in females. CMI correlated positively with grip strength (*ρ* = 0.47 [0.43, 0.50] and 0.45 [0.40, 0.49]) and negatively with TUG (*ρ* = –0.37 [−0.41, −0.32] and −0.44 [−0.49, −0.40]) in males and females, respectively. In adjusted models, the HR for mortality per 100 mg/day increase in CMI was HR 0.85 (95% CI 0.78, 0.90 *p* value < 0.001) in males and HR 0.77 (95% CI 0.67, 0.87 *p* value < 0.001) in females. In males and females, respectively, the C-index of CMI for probable sarcopenia (0.73 and 0.71) and mortality (0.70 and 0.76) was higher than that of the eGFR ratio (probable sarcopenia: 0.64 and 0.61; mortality: 0.60 and 0.65; all *p* < 0.001) and the eGFR difference (probable sarcopenia: 0.59 and 0.57; mortality: 0.56 and 0.59; all *p* < 0.001) Limitations include the observational design, the assessment of muscle function without direct measurement of muscle mass, and limited generalisability to CKD populations not followed in secondary care.

**Conclusions:**

In persons with CKD, CMI—a biomarker reflecting creatinine generation—was independently associated with muscle function and mortality, supporting its utility in populations with reduced kidney function.

## Author summary

**Why was this study done?:**

- Loss of muscle mass and strength (sarcopenia) is common in people with chronic kidney disease (CKD) and is linked to a higher risk of death and poorer quality of life.
- Creatinine is a marker of kidney function, but is metabolised in muscle. Cystatin C is a measure of kidney function that is independent of muscle mass. Blood tests comparing cystatin C and creatinine have been used to estimate muscle mass, but they have been understudied and less accurate in people with CKD.
- The Creatinine Muscle Index (CMI) is a newer measure that better reflects muscle-related creatinine production. Its usefulness in people with CKD had not been adequately tested.

**What did the researchers do and find?:**

- The study included 2,930 adults with non-dialysis CKD from 16 kidney centres across the United Kingdom. Participants had their CMI and muscle function (grip strength and walking speed) measured and were followed for a median of 50 months.
- In both men and women, lower CMI (indicating lower muscle mass) was linked to weaker hand grip strength, slower walking speed, and a higher risk of sarcopenia.
- Higher CMI was also linked to a lower risk of death. For every 100 mg/day per 1.73 m^2^ increase in CMI, the risk of death fell by 15% in men and 23% in women. CMI outperformed other cystatin C-creatinine-based measures in predicting mortality and sarcopenia.

**What do these findings mean?:**

- Because kidney disease affects how creatinine is processed, it was uncertain whether CMI would work well in people with CKD. This study shows that CMI remains independently associated with both muscle function and survival in this population.
- Given the high rates of sarcopenia in CKD, CMI may be a useful tool to help identify people at higher risk of muscle loss and its related complications, supporting better monitoring and potential early interventions.
- The main limitations are that this was an observational study and muscle strength was measured, but muscle mass itself was not directly assessed

## Abstract 3

Thomas McDonnell and colleagues examine the relationship of creatinine muscle index (CMI), an estimate of glomerular filtration of creatinine, with sarcopenia and mortality in a non-dialysis chronic kidney disease population.

## Introduction

Sarcopenia is increasingly recognised as a significant risk factor for adverse health outcomes and is now formally classified as a distinct disease entity [1–3]. It was defined by the European Working Group on Sarcopenia in Older People (EWGSOP) as a progressive and generalised skeletal muscle disorder that is associated with an increased risk of falls, fractures, physical disability, and mortality [4]. While initially described as an age-related decline in muscle mass and function [5], sarcopenia is also recognised to affect younger populations suffering from chronic diseases such as diabetes, cancer, cardiovascular, and kidney disease [6,7].

Chronic kidney disease (CKD) results in a metabolic environment that promotes the development of sarcopenia [8], with a higher prevalence than in the general population and age-matched controls [9]. A meta-analysis of 140 studies estimated the prevalence of sarcopenia in non-dialysis CKD at 19.6%, with no significant variation across CKD stages [10]. Sarcopenia in CKD is strongly associated with increased mortality, emphasising the importance of detection [9,11].

Given the high prevalence and strong link to mortality, finding simple biomarkers to identify those at risk of sarcopenia and death in CKD is crucial so that interventions to improve physical performance can be introduced. While creatinine generation decreases with decreasing muscle mass, serum creatinine concentration alone is of limited use in individuals with impaired kidney function, as serum creatinine levels increase as the glomerular filtration rate (GFR) declines. 24-hour urinary creatinine excretion is accepted as a surrogate for muscle mass, but it is prone to measurement inaccuracies and logistical challenges [12–14].

The Creatinine Muscle Index (CMI) is a surrogate marker designed to estimate glomerular filtration of creatinine and creatinine generation from muscle [15]. By using cystatin C, as an alternative method for estimating GFR (eGFRcys) that is independent of muscle mass [16], it is possible to estimate the muscle-dependent component of serum creatinine concentration.

CMI has been previously examined in two important studies. The first, conducted in an older population, demonstrated an association with mortality and frailty [15]. The second, involving a smaller cohort, identified associations with measures of muscle function and mass (thigh muscle area on CT scan); however, only a subset of participants had CKD, and no mortality data were available [17]. We hypothesised that higher CMI is associated with a decreased risk of all-cause mortality in a large, prevalent, non-dialysis CKD cohort and that CMI is positively associated with measures of muscle function.

## Methods

### Study design

The National Unified Renal Translational Research Enterprise (NURTuRE)-CKD is an ongoing UK, prospective multicentre cohort study of 2,996 adults with an eGFR of 15–59 mL/min/1.73 m^2^ or eGFR ≥60 mL/min/1.73 m^2^ with a urine albumin-to-creatinine ratio (uACR) >30 mg/mmol. Recruitment from 16 nephrology centres in England, Wales, and Scotland was completed in 2019. The study design and methods of NURTuRE-CKD have been previously reported [18], including anthropometric, muscle function, and health-related quality-of-life measures [19]. The study was conducted according to a prospective analysis plan that was developed prior to data analysis. This plan is provided as a Supporting Information file (S1 File). CKD status was defined based on a single set of eGFR and albuminuria measurements rather than requiring confirmation at least 3 months apart, as recommended by KDIGO guidelines. However, all participants were recruited from specialist nephrology clinics with confirmed CKD and underwent repeat centralised measurements at study enrolment; none were excluded for failing to meet KDIGO CKD criteria.

### Ethics

All participants provided written informed consent. The study was approved by the South Central—Berkshire Research Ethics Committee, abides by the principles of the Declaration of Helsinki (IRAS project ID: 211479) and is registered at ClinicalTrials.gov (NCT04084145) and includes the current study presented in this manuscript.

### Laboratory methods

Demographic and clinical data were obtained at baseline visits at which blood and urine samples were collected and stored in a biorepository according to standard operating procedures that required plasma and serum samples to be frozen within 2 hours of collection. Serum creatinine, uACR, cystatin C (Tina-quant Cystatin C Gen 2 method from Roche, IFCC standardisation against ERM-DA471 reference material [20]), and C-Reactive Protein (CRP) were analysed in stored samples on routine Roche Cobas 8000/c702/c502 chemistry analysers under ISO 15189 certification at Geneva University Hospitals, Switzerland. The intra- and inter-assay coefficients of variation of the cystatin C assay were assessed on results from 84 separate samples. Creatinine-based estimated GFR (eGFRcr) was calculated via the 2009 Chronic Kidney Disease Epidemiology Collaboration equation without the ethnicity variable, as recommended by the National Institute for Health and Care Excellence in 2021 [21]. Cystatin C-based estimated GFR (eGFRcys) was calculated via the 2012 Chronic Kidney Disease Epidemiology Collaboration (CKD-EPI) cystatin C equation [22], and eGFRcr-cys was calculated using the 2021 CKD-EPI equation [23].

### CMI explanation and calculation

Serum creatinine concentration reflects the balance between creatinine generation from muscle metabolism, dietary intake, and urinary excretion. Based on an understanding of this physiology, Ballew and colleagues proposed the use of the CMI [15], an estimate of the glomerular filtration of creatinine calculated as the product of serum creatinine concentration and GFR, as a surrogate biomarker of muscle mass. To avoid confounding of the estimated GFR by serum creatinine concentration, they proposed the use of GFR estimated from serum cystatin C concentration, which is not related to muscle mass. Evidence that CMI is a valid biomarker of muscle mass has been provided by studies showing significant associations between CMI and measures of frailty as well as muscle mass (thigh muscle area on CT scan) and muscle function [15–17]. The CMI differs from other indices reflecting differences in GFR estimated from creatinine versus cystatin C (such as the ratio or difference) by being grounded in the underlying physiology rather than mathematics. The CMI was calculated as: eGFRcys (ml/min per 1.73 m^2^) × serum creatinine (mg/dl) × 1 dl/100 ml × 1,440 min/day, reported in mg/day per 1.73 m^2^ [15].

### Analysis plan

**Cross-sectional analysis:** The relationship of CMI to sarcopenia at baseline, defined by the EWGSOP2 criteria of best hand grip strength of <27 kg for males and <16 kg for females was examined [4]. EWGSOP2 defines sarcopenia as ‘probable’ when direct measures of muscle mass are not available.

The relationship of CMI to functional measures as continuous variables. (1) best hand grip strength and( 2) the timed get-up-and-go (TUG) test, an assessment of how long it takes an individual to stand up from a chair, walk 3 m, turn, walk back, and sit down. Additionally, functional status was assessed using the Karnofsky Performance Score (KPS) [24,25] on a scale of 0 (dead) to 100 (normal or no complaints) at baseline. Health-related quality-of-life (HRQoL), calculated by the 5-level EuroQol Five Dimensions (EQ-5D-5L) [26], provides an overall EQ-5D-5L index value, which was converted to the EQ-5D-3L index value for analysis, as suggested by NICE [19]. Values ranged from 1 (perfect health) to 0 (equivalent health status to death) or negative values (health status worse than death) with a scoring range of −0.594 to 1.

**Longitudinal analysis endpoint:** All-cause mortality before initiation of kidney replacement therapy (KRT) defined by dialysis (either haemodialysis or peritoneal dialysis) or transplantation. Participants were censored at the time of KRT initiation as per the study protocol (NCT04084145). Including mortality events after KRT would introduce heterogeneity, as the risk factors and determinants of death in dialysis patients differ markedly from those in pre-dialysis CKD. Moreover, the initiation of dialysis is accompanied by rapid muscle loss that would not be captured by baseline CMI, thereby obscuring the relationship between baseline CMI and subsequent mortality. Data regarding the date of death and KRT was obtained from the UK Renal Registry. Participants were censored at the time of death, the initiation of KRT, or the last follow-up on 31/12/2022.

This study is reported as per the Strengthening the Reporting of Observational Studies in Epidemiology (STROBE) guideline (S1 Checklist).

### Statistical methods

The cohort was split by sex as men are known to have greater muscle mass and creatinine production than women [27,28]. Sex-specific tertiles were created, and differences in anthropometric measures, demographics, comorbidities, disease markers, sarcopenic measures, HRQoL metrics, and mortality were examined. Continuous variables are expressed as median (25th percentile [Q1] to 75th percentile [Q3]), with significance testing using the Kruskal-Wallis rank sum test. Categorical variables are expressed as numbers (%) with the Chi-squared test. Using the gtsummary package in R.

Probable sarcopenia was assessed as a binary outcome (Yes/No) using the sex-specific cut-offs for low hand grip strength, according to the EWGSOP2 criteria. Logistic regression models were used to evaluate the association between CMI and outcome measures at baseline. Cox proportional hazards models were used to assess the relationship of CMI with time to all-cause mortality using the R survival package. The correlation of CMI to individual functional measures of grip strength, TUG, EQ-5D-3L, and KPS was assessed via linear regression and Spearman’s correlation coefficient (due to right-skewed data).

Both unadjusted and adjusted regression models were fitted. Covariates included in the adjusted model were selected a priori based on the univariable analyses and their potential role as confounders of associations between CMI, probable sarcopenia, and mortality. Adjustments were made for the following confounders: White ethnicity, age, body mass index (BMI), smoking all of which are associated with increased risk of sarcopenia and death [29–32]. Charlson Comorbidity Index (CCI) is a validated scoring system that predicts the risk of long-term mortality based on comorbidities [33], with higher scores increasing risk of sarcopenia [34]. The components of the CCI largely reflect chronic disease processes that typically precede changes in muscle mass and creatinine generation, through mechanisms such as inflammation, reduced physical activity, and catabolic states. Urine ACR is a marker of CKD, a known risk factor for sarcopenia and higher mortality. CRP, a biomarker of inflammation, is also associated with sarcopenia and increased risk of death [35]. Missing data were addressed for all covariates used in regression models. Where missingness was minimal (<4%), median or mode imputation was applied. Where missingness was greater (4%–10%), regression-based imputation was used (for uACR, eGFR was used as the predictor). The Schoenfeld residuals test for proportional hazards was used to check the proportional hazards assumption and whether the effect of CMI on the hazard ratio (HR) was proportional over time Due to the differences in risk of both sarcopenia and death at extremes of age and BMI the associations of CMI with mortality and probable sarcopenia were also explored in subgroups of age (<65 versus ≥65 years) and BMI (<30 versus ≥30 kg/m^2^).

A restricted cubic spline model with three knots was employed to assess the linearity of the relationship between CMI and mortality before KRT. This analysis was presented both as an unadjusted and adjusted model, using the same adjustments described above, using the splines package in R.

In both Cox proportional hazards models and in logistic and linear regression analyses, CMI was scaled to 100 mg/day per 1.73 m^2^ to enhance interpretability. The results are presented as the change in risk or coefficient for every 100 mg/day per 1.73 m^2^ increase or decrease in CMI, providing an intuitive, unit-based estimate. However, as a 100 mg/day per 1.73 m^2^ change represents a relatively different proportional change between males and females, Cox and logistic regression models were repeated with CMI expressed per one standard deviation (SD) increase in the biomarker to facilitate meaningful comparisons across sexes. Scatter plots were used to illustrate the relationships between CMI and grip strength and TUG. Because CMI was right-skewed, it was analysed on the natural-log scale; for ease of interpretation, the x-axis was back-transformed and displayed in its original units (mg/day per 1.73 m^2^).

CMI was compared to other equations of cystatin C-creatinine-based differences: eGFR ratio (eGFRcys/eGFRcr) and eGFR difference (eGFRcys − eGFRcr), in addition to age and BMI combined for predicting probable sarcopenia. These, in addition to hand grip strength as a continuous variable and at the EWGSOP2 cut-off, were compared to sarcopenia for the prediction of mortality. The discriminatory power of each equation was calculated using the area under the curve (AUC) for probable sarcopenia and Harrell’s C-Index for mortality. AUCs were compared with DeLong’s test from pROC package in R and C-indices with the paired U-statistic from the compareC R packages, respectively.

The analyses performed were consistent with the pre-specified analytical plan above, and no deviations or unplanned analyses were undertaken All statistical analysis was performed using R Studio version 2023.09.1.

## Results

Of the 2,996 adults enrolled in NURTuRE-CKD, 2,930 (1,723 males, 1,207 females) had cystatin C levels measured at baseline. The flow chart of exclusion and available data are shown in S1 Fig. S1 Table details the number (%) of missing values for each covariate included in the regression analyses.

The intra-assay variation coefficients, which assess precision within the same assay for cystatin C, were 0.7%, 0.9%, and 1.7% for high, medium, and low concentrations, respectively. The inter-assay variation coefficients, which measure precision across different assay runs, were 2.2%, 1.4%, and 1.4% for the corresponding concentration levels. These results indicate good analytical precision for cystatin-C across all concentration ranges.

Table 1 presents the baseline characteristics of the cohort, stratified by sex and CMI tertiles. At baseline, the median (IQR) age of the overall cohort was 66 (53 to 74) years, eGFRcr 34 (24 to 47) ml/min/1.73 m^2^, eGFRcys 29 (21 to 40) ml/min/1.73 m^2,^ and uACR 23 (4 to 105) mg/mmol. Probable sarcopenia was present in 806 (28%). In those with probable sarcopenia at baseline versus those without, EQ-5D indices were lower at 0.79 (0.54 to 0.92) versus 0.89 (0.78 to 1.00) (*p* value < 0.001), as were Karnofsky scores 80 (70–90) versus 90 (80–100) (*p* value < 0.001) and mortality rates were higher at 34% compared to 12% (*p* value < 0.001) over the 50 months median follow-up time.

**Table 1:** Baseline data in subgroups defined by sex-specific tertiles of Creatinine Muscle Index (CMI).

Male				Female			
Number	575	574	574	Number	403	402	402
CMI (*mg/day per 1.73 m*^*2*^) (tertiles at baseline)	675 (616 to 727)	864 (820 to 911)	1,094 (1,020 to 1,232)	CMI (*mg/day per 1.73 m*^*2*^)	541 (479 to 586)	704 (668 to 745)	914 (841 to 1,045)
**Demographics and Anthropometrics**							
Age (years)	73 (68 to 79)	68 (59 to 74)	55 (44 to 65)	Age (years)	71 (64 to 78)	65 (55 to 73)	50 (39 to 60)
White ethnicity	524 (91%)	513 (89%)	472 (82%)	White ethnicity	375 (93%)	355 (88%)	336 (84%)
Smoker	361 (63%)	304 (53%)	250 (44%)	Smoker	199 (50%)	170 (43%)	158 (40%)
BMI (kg/m2)	29.0 (25.3 to 33.5)	28.4 (25.8 to 31.9)	28.7 (25.6 to 31.9)	BMI	30 (26 to 35)	28 (25 to 33)	27 (24 to 32)
Waist/hip ratio	0.99 (0.95 to 1.04)	0.99 (0.95 to 1.03)	0.97 (0.93 to 1.02)	Waist/hip ratio	0.90 (0.85 to 0.96)	0.89 (0.83 to 0.94)	0.86 (0.81 to 0.91)
Waist/ height ratio	0.61 (0.56 to 0.68)	0.60 (0.56 to 0.66)	0.58 (0.53 to 0.63)	Waist/height Ratio	0.64 (0.56 to 0.71)	0.60 (0.54 to 0.67)	0.56 (0.49 to 0.62)
**Comorbidities**							
Diabetes	266 (47%)	201 (36%)	114 (20%)	Diabetes	155 (39%)	108 (28%)	56 (15%)
Hypertension	498 (87%)	496 (88%)	494 (89%)	Hypertension	340 (85%)	321 (82%)	304 (79%)
CABG	49 (8.6%)	21 (3.7%)	10 (1.8%)	CABG	9 (2.3%)	7 (1.8%)	4 (1.0%)
MI	102 (18%)	76 (13%)	32 (5.7%)	MI	32 (8.0%)	24 (6.1%)	6 (1.6%)
Stroke	33 (5.8%)	23 (4.1%)	14 (2.5%)	Stroke	17 (4.3%)	19 (4.8%)	4 (1.0%)
PVD	45 (7.9%)	29 (5.1%)	11 (2.0%)	PVD	16 (4.0%)	17 (4.3%)	3 (0.8%)
AF	88 (15%)	69 (12%)	33 (5.9%)	AF	53 (13%)	29 (7.4%)	10 (2.6%)
CCI score	5.00 (4.00 to 6.00)	4.00 (2.00 to 5.00)	2.00 (1.00 to 4.00)	CCI score	4.00 (3.00 to 5.00)	3.00 (2.00 to 4.00)	1.00 (0.00 to 3.00)
**Biomarkers**							
Urine ACR (mg/mmol)	40 (6 to 128)	33 (6 to 119)	26 (4 to 107)	ACR mg/mmol	17 (3 to 81)	10 (2 to 62)	11 (2 to 78)
CRP (mg/l)	3.8 (1.5 to 9.1)	2.4 (1.1 to 5.1)	1.7 (0.8 to 3.8)	CRP mg/l	3.9 (1.9 to 7.2)	2.4 (1.1 to 5.0)	1.8 (0.8 to 3.9)
eGFRcr mL/min/1.73 m^2^ (2009 CKD-EPI)	32 (22 to 42)	32 (23 to 43)	35 (24 to 49)	eGFRcr (2009 CKD-EPI)	33 (25 to 43)	35 (25 to 48)	41 (27 to 55)
eGFRcys mL/min/1.73 m^2^ (2012 CKD-EPI)	22 (17 to 29)	29 (22 to 36)	37 (28 to 51)	eGFRcys (2012 CKD-EPI)	23 (18 to 29)	32 (24 to 41)	43 (31 to 58)
eGFRcr-cys mL/min/1.73 m^2^ (2021 CKD-EPI)	25 (19 to 34)	30 (22 to 39)	36 (25 to 50)	eGFRcr-cys (2021 CKD-EPI)	24 (19 to 32)	30 (22 to 39)	37 (25 to 51)
**Sarcopenic to functional and QOL measures at baseline**							
Probable Sarcopenia	257 (45%)	144 (25%)	53 (9.2%)	Sarcopenia Y/N	188 (47%)	118 (29%)	46 (11%)
Grip strength (kg)	28 (23 to 33)	32 (27 to 39)	38 (33 to 44)	Grip strength (kg)	16 (12 to 20)	19 (15 to 23)	23 (19 to 28)
Timed up-and-go (seconds)	11.1 (8.9 to 14.0)	9.5 (8.0 to 11.6)	8.4 (7.0 to 10.0)	Timed up and go (seconds)	12.0 (9.3 to 18.1)	9.7 (8.0 to 12.6)	8.1 (6.9 to 10.0)
EQ-5D-3L index	0.83 (0.65 to 0.94)	0.92 (0.78 to 1.00)	0.94 (0.83 to 1.00)	EQ-5D-3L index	0.75 (0.51 to 0.88)	0.84 (0.67 to 1.00)	0.92 (0.79 to 1.00)
KPS	90 (70 to 100)	90 (80 to 100)	100 (90 to 100)	KPS	80 (70 to 90)	90 (80 to 100)	100 (90 to 100)
**Mortality**							
Follow-up time (months)	45.5 (29.2 to 53.5)	49.5 (41.4 to 55.6)	51.4 (42.4 to 57.0)	Follow-up time (months)	48.0 (37.2 to 54.4))	51.9 (44.7 to 56.5)	53.3 (47.5 to 57.5)
All-Cause Mortality	206 (36%)	94 (16%)	52 (9.1%)	All-Cause Mortality	115 (29%)	46 (11%)	14 (3.5%)

For continuous variables, results are reported as median (IQR); for categorical variables, as *n* (%). CMI (Creatinine Muscle Index), BMI (Body Mass Index), ‘smoker’ includes those who self-reported current or previous tobacco cigarette consumption, BMI (body mass index), CABG (Coronary Artery Bypass Graft), MI (Myocardial Infarction), PVD (Peripheral Vascular Disease), AF (Atrial Fibrillation), and CCI (Charlson Comorbidity Index). ACR (Albumin-Creatinine Ratio), CRP (C-Reactive Protein), EQ-5D-3L is a standardised measure of health-related quality-of-life. Values ranged from 1 (perfect health) to 0 (equivalent health status to death) or negative values (health status worse than death). KPS (Karnofsky Performance Score) is a measure of functional status, scaled from 0 (dead) to 100 (normal or no complaints). Sarcopenia Yes/NO (Y/N) defined by the best hand grip strength of <27 kg for males and <16 kg for females, based upon the European Working Group on Sarcopenia in Older People 2 (EWGSOP2) criteria. Significance: Kruskal–Wallis rank sum test (continuous variables)and the Chi-squared test (categorical).

The distribution of CMI was similar for males and females (S2 Table). However, the median CMI was higher for males than for females, at 864 (727 to 1,020) mg/day per 1.73 m^2^, compared to 704 (586 to 841) mg/day per 1.73 m^2^ (S2 Table). CMI was split into sex-specific tertiles (Table 1). For both males and females, age and all co-morbidities (except hypertension), CRP, and uACR increased in the lower tertiles of CMI. There was a higher proportion of self-reported non-white ethnicity in the highest CMI tertile. BMI increased across the lower tertiles of CMI only in women; however, waist/hip and waist/height ratios were lower in the highest CMI tertile for men and women.

For both men and women, rates of probable sarcopenia increased with decreasing CMI. In men, 45% appeared to be sarcopenic in the lowest CMI tertile versus 9.2% in the highest tertile. In women, 47% appeared to be sarcopenic in the lowest CMI tertile versus 11% in the highest tertile. The odds of probable sarcopenia, for every 100 mg/day per 1.73 m^2^ increase in CMI, are shown in Table 2. In unadjusted analysis, the odds ratio (OR; 95% confidence interval [CI]) for males was OR 0.63 (95% CI 0.59,0.68; *p* value <0.001) and for females, OR was 0.65 (95% CI 0.60, 0.71; *p* value <0.001). In the adjusted analysis, the OR was 0.72 (95% CI 0.67, 0.78; *p* value <0.001) in males and OR was 0.81 (95% CI 0.73, 0.89; *p* value < 0.001) in females. Hence, in the fully adjusted model, for every 100 mg/day per 1.73 m^2^ increase in CMI the odds of sarcopenia decreased by 28% in males and 19% in females. The direction and magnitude of associations were consistent when comparing the ORs of males and females for sarcopenia per SD change in log-transformed CMI (S3 Table). CMI remained significantly associated with probable sarcopenia in all subgroups (Age <65 versus ≥65 years, BMI <30 versus ≥30 kg/m^2^) (S4 Table).

**Table 2:** Association of Creatinine Muscle Index (per 100 mg/day increase) with probable sarcopenia, defined by low grip strength.

	Unadjusted		Adjusted	
	OR (95% CI)	*P* value	OR (95% CI)	*P* value
**Male**	0.63 (0.59, 0.68)	<0.001	0.72 (0.67, 0.78)	<0.001
**Female**	0.65 (0.6, 0.71)	<0.001	0.81 (0.73, 0.89)	<0.001

Low grip strength was defined as <27 kg for males and <16 kg for females, consistent with the European Working Group on Sarcopenia in Older People 2 (EWGSOP2) definition of probable sarcopenia. Odds ratios (ORs) were estimated using logistic regression to assess the association between Creatinine Muscle Index (CMI) and sarcopenia defined by low grip strength at baseline. ORs are reported per 100 mg/day per 1.73 m^2^ increase in CMI. Adjustments are for age, white ethnicity, body mass index, smoking status, Charlson Comorbidity Index, urinary albumin-to-creatinine ratio (uACR), and C-reactive protein (CRP).

**Fig 1:**
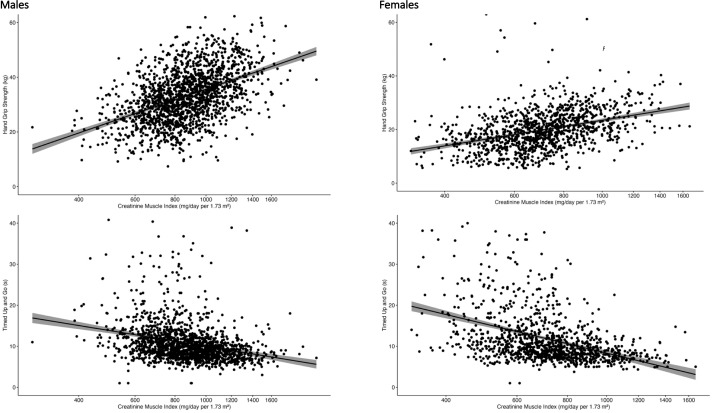
Scatter plots for log-transformed Creatinine Muscle Index (CMI), hand grip strength, and timed get-up-and-go (TUG) in males and females. Scatter plots illustrate the relationship between CMI (mg/day per 1.73 m^2^)—shown on the x-axis as values that have been back-transformed from their natural-log form for easier interpretation—and best hand grip strength (kg) and TUG performance in females and males. Each point represents an individual participant, and the solid black line depicts the fitted linear-regression trend.

Both grip strength and TUG worsened across the lower CMI tertiles; the correlation of CMI with measures of muscle function can be seen in Table 3 and Fig 1. In both men and women, increasing CMI was positively correlated with grip strength and negatively correlated with TUG. In linear regression models, the relationship persisted after multiple adjustments for each functional measure. However, after adjustment, the association observed for TUG in males was attenuated (−0.14 (−0.29, −0.02), *p* value 0.049). In both males and females, the strongest correlation was with grip strength (Spearman’s rho correlation (*ρ*) 0.47 (95% CI 0.43,0.50) and *ρ* 0.45 (95% 0.40,0.49), respectively).

**Table 3:** Associations of creatine muscle index with measures of muscle function, functional performance, and health-related quality-of-life.

	Male					Female				
Outcome	*ρ* (95% CI)	Unadjusted *β*	*P* value	Adjusted *β*	*P* value	*ρ* (95% CI)	Unadjusted *β*	*P* value	Adjusted *β*	*P* value
**Best hand grip strength (kg)**	0.47 (0.43 to 0.50)	1.92 (1.74, 2.79)	<0.001	1.13 (0.92, 1.34)	<0.001	0.45 (0.40 to 0.49)	1.41 (1.21, 1.60)	<0.001	0.65 (0.41, 0.89)	<0.001
**Timed get-up-and-go (s)**	−0.37 (−0.41 to −0.32)	−0.56 (−0.68, −0.44)	<0.001	−0.14 (−0.29, −0.02)	0.049	−0.44 (−0.49 to −0.40)	−1.27 (−1.46, −1.08)	<0.001	−0.63 (−0.86, −0.39)	<0.001
**EQ-5D-3L**	0.29 (0.25 to 0.34)	0.02 (0.02, 0.03)	<0.001	0.01 (0.01, 0.02)	<0.001	0.31 (0.25 to 0.36)	0.03 (0.03, 0.04)	<0.001	0.02 (0.01, 0.03)	<0.001
**KPS**	0.31 (0.27 to 0.35)	1.63 (1.33, 1.92)	<0.001	0.80 (0.44, 1.15)	<0.001	0.31 (0.26 to 0.36)	2.15 (1.72, 2.58)	<0.001	1.20 (0.67, 1.73)	<0.001

*ρ* (95% confidence interval [CI]) represents Spearman’s rho correlation. Regression coefficient (*β*) and corresponding 95% CI, indicate the change in the outcome for each 100 mg/day per 1.73 m^2^ increase in CMI. Β’s are presented as unadjusted and adjusted for age, white ethnicity, body mass index, smoking status, the Charlson Comorbidity Index, urinary albumin-to-creatinine ratio, and C-reactive protein. Best hand grip strength is measured in kg and the timed get-up-and-go test is an assessment of how long it takes an individual to stand up from a chair, walk 3 m, turn, walk back, and sit down. The EQ-5D-3L measures quality of life as a standardised health-related quality-of-life measure, with values ranging from 1 (perfect health) to 0 (health status equivalent to death) or negative values (health status worse than death). KPS (Karnofsky Performance Score) is a measure of functional status, scaled from 0 (dead) to 100 (normal or no complaints).

Regarding functional status, there was a significant positive relationship between increasing CMI and KPS, with adjusted *β* values of 0.80 (95% CI 0.44, 1.55; *p* value <0.001) for men and *β* 1.20 (95% CI 0.67, 1.73; *p* value <0.001) for women. Increasing CMI was also associated with a statistically significant increase in health-related QoL in adjusted models with *β* values of 0.01 (95% CI 0.01, 0.02; *p* value <0.001) and 0.02 (95% CI 0.01, 0.03; *p* value < 0.001) in males and females, respectively. However, the magnitude of this association may not be clinically meaningful.

The median follow-up time was 50 (41 to 56) months, during which 527 (18%) participants died pre-KRT. Mortality rates were highest in the lowest tertile of CMI and progressively decreased across tertiles for both sexes. In males, mortality rates were 36% in the lowest tertile versus 9.1% in the highest tertile. Similarly, in females, mortality rates were 29% versus 3.5%.

Cox proportional hazards models were constructed to assess the associated risk of mortality pre-KRT in both men and women (Table 4). In unadjusted analysis, the HR and 95% confidence intervals for mortality pre-KRT per every 100 mg/day per 1.73 m^2^ increase in CMI were HR 0.68 (95% CI 0.64,0.72; *p* value <0.001) for males and HR 0.54 (95% Ci 0.49, 0.60; *p* value <0.001) for females. In adjusted models, the HR was 0.85 (95% CI 0.78, 0.90; *p* value <0.001) in males and HR 0.77 (95% CI 0.67, 0.87; *p* value <0.001) in females. Hence, for every 100 mg/day per 1.73 m^2^ increase in CMI, the risk of mortality decreased by 15% for males and 23% for females. The direction and magnitude were similar when analysing the risk of death per SD change in log-transformed CMI (S5 Table). The association of CMI remained significant in all subgroups (Age <65 versus ≥65 years, BMI <30 versus ≥30) kg/m^2^) (S6 Table). The Schoenfeld residuals test *p* value for proportional hazards was 0.97; hence, the effect of CMI on the HR was consistent over time, respecting the proportionality principle.

**Table 4:** Associations of Creatinine Muscle Index (per 100 mg/day increase) with All-Cause Mortality.

	Unadjusted		Adjusted	
	HR (95% CI)	*P* value	HR (95% CI)	*P* value
**Male**	0.68 (0.64, 0.72)	<0.001	0.85 (0.78, 0.90)	<0.001
**Female**	0.54 (0.49, 0.60)	<0.001	0.77 (0.67, 0.87)	<0.001

Hazard ratios (HRs) were estimated using Cox proportional hazards regression to assess the association between CMI and all-cause mortality prior to the initiation of kidney replacement therapy, defined as dialysis or kidney transplantation. HRs are reported per 100 mg/day per 1.73 m^2^ increase in CMI. Adjustments are for age, white ethnicity, body mass index, smoking status, Charlson Comorbidity Index, urinary albumin-to-creatinine ratio, and C-reactive protein.

In the unadjusted restricted cubic spline model (S3 Fig), there appeared to be a linear relationship between CMI and the log relative hazard of mortality. In the adjusted analysis (S4 Fig), the relationship between CMI and the log relative hazard of mortality (and hence the protective effect) appeared to plateau as CMI increased. Both unadjusted and adjusted restricted cubic splines were statistically significant.

Table 5 compares the discriminative performance of CMI with that of other cystatin C and creatinine indices, as well as anthropometric and functional measures. The C-statistics showed that, among males, CMI was a stronger predictor of mortality than sarcopenia (defined as hand grip strength <27 kg), the eGFR ratio, and the eGFR difference. Its predictive performance was comparable to that of grip strength when treated as a continuous variable. In females, CMI outperformed all other metrics. For predicting probable sarcopenia, CMI demonstrated superior discrimination compared with both eGFR-based measures in men and women. When CMI was compared to age and BMI, it had comparable discrimination for probable sarcopenia. When CMI was added to age and BMI, it resulted in significantly improved discrimination in males (AUC 0.77 [95% CI: 0.74, 0.79] versus 0.73 [95% CI 0.70, 0.75]; *p* value <0.001) and in females (AUC 0.74; [95% CI: 0.71, 0.77] versus 0.72 [95% CI 0.68, 0.75]; *p* value 0.006).

**Table 5:** Comparing the discrimination of different metrics for predicting mortality and probable sarcopenia.

	Male						Female					
	Mortality			Probable Sarcopenia			Mortality			Probable Sarcopenia		
	C index	95% CI	*P* value^*^	AUC	95% CI	*P* value^*^	C index		*P* value^*^	AUC		*P* value^*^
CMI	0.70	0.67, 0.73	NA	0.73	0.71, 0.76	NA	0.76	0.71, 0.80	N.A	0.71	0.68, 0.74	NA
eGFRdiff	0.56	0.53, 0.59	<0.001	0.59	0.57, 0.62	<0.001	0.59	0.55, 0.64	0.001	0.57	0.54, 0.61	<0.001
eGFR ratio	0.60	0.57, 0.63	<0.001	0.64	0.61, 0.67	<0.001	0.65	0.61, 0.70	0.021	0.61	0.58, 0.65	<0.001
Probable Sarcopenia	0.64	0.62, 0.66	0.009	NA	NA	NA	0.67	0.64, 0.70	0.02	NA	NA	NA
Grip Strength	0.71	0.67, 0.74	0.254	NA	NA	NA	0.71	0.67, 0.76	0.043	NA	NA	NA
Age + BMI	0.72	0.68, 0.75	<0.001	0.73	0.70, 0.75	0.539	0.77	0.73, 0.82	<0.001	0.72	0.68, 0.75	0.73

CMI (creatine muscle index), eGFR ratio (eGFRcys/eGFRcr), and eGFR difference (eGFRcys − eGFRcr); Probable Sarcopenia defined by the best hand grip strength (HGS) of <27 kg for males and <16 kg for females, based on the European Working Group on Sarcopenia in Older People 2 (EWGSOP2) criteria. Discrimination for mortality was assessed using Harrell’s concordance statistic (C-statistic) and its 95% confidence interval (CI), and sarcopenia was assessed using the area under the curve (AUC).

* *P* values test whether each metric’s (C-index or AUC) differs from that of CMI (paired comparison, for C-indices and DeLong’s test for AUCs). The C index for age alone for mortality was 0.71 in males and 0.76 in females. The AUCs for age, BMI, and CMI in males was 0.77 (95% CI: 0.74, 0.79), *p* = < 0.001 and in females 0.74 (95% CI: 0.71, 0.77), *p* = 0.006. *P value for comparison to age and BMI.*

## Discussion

In this large, multicentre prospective cohort study of people with non-dialysis CKD, we observed a high prevalence of sarcopenia, which was associated with increased mortality, poorer self-reported HRQoL, and reduced functional status. We demonstrated that CMI, an estimator of creatinine filtration and muscle-derived creatinine generation, was associated with baseline markers of sarcopenia and to all-cause mortality. Although indices based on creatinine- and cystatin C-derived eGFR (eGFRdiff and eGFRratio) have been proposed for this purpose, their accuracy is limited at lower levels of kidney function. Our findings support the use of CMI in individuals with reduced eGFR, highlight its superiority over these alternative metrics, and suggest its potential as a blood-based biomarker of sarcopenia and mortality risk.

Definitions of sarcopenia have varied over time [9,36], with earlier criteria relying heavily on direct measures of muscle mass that are difficult and costly to obtain in routine practice [37]. In 2019, the EWGSOP2 redefined sarcopenia as ‘muscle failure’, prioritising muscle strength and function over muscle mass [4] because of their greater practicality and stronger ability to predict adverse outcomes [38–41]. Our study aligns with this paradigm shift by focussing on functional measures (hand grip strength and TUG).

In this study, CMI correlated positively with grip strength and negatively with TUG, two established markers of muscle function. These findings align with a study by Oka and colleagues involving 749 older Icelanders, in which CMI showed similar correlations with grip strength and TUG, though only 40% had CKD [17]. That study also calculated CMI using measured GFR and found comparable associations with muscle function. Notably, they assessed muscle mass via thigh CT, observing moderately strong correlations with both estimated and measured CMI, though stronger for the latter. In addition, a smaller study of 297 Taiwanese patients with non-dialysis CKD reported significant correlations between CMI and measures of muscle mass assessed by bioimpedance, as well as muscle function assessed by HGS and TUG, with correlation coefficients comparable to those observed in our study [42]. Our findings, therefore, confirm and extend prior observations by demonstrating consistent associations between CMI and muscle function in a substantially larger, multicenter cohort of patients with non-dialysis CKD, using centralised laboratory measurements.

Our findings also demonstrate that, after adjusting for confounders, lower CMI was strongly associated with an increased risk of mortality in a non-dialysis CKD population. This association persisted in subgroups of low and high BMI and age. Prior studies have examined indices derived from serum creatinine and cystatin C-based eGFR, demonstrating associations with markers of muscle mass, function, and mortality [16]. However, approximately 75% of these studies were conducted in Asian populations, and the indices tended to perform poorly in individuals with reduced eGFR. While a small number of studies have specifically evaluated these indices in prevalent CKD populations [43–47], most focussed on the eGFR ratio and eGFR difference, and few included both muscle-function and mortality outcomes. One recent study of 1,290 people with CKD examined associations of eGFR difference with mortality and measures of muscle mass. While eGFR difference was strongly linked to mortality and unaffected by adjustments for putative determinants of eGFR, the association with muscle mass was weak [48].

It may be that the eGFR difference and ratio are inferior to CMI because CMI is physiologically linked to creatinine generation, rather than being solely a mathematical construct. In support of this, CMI outperformed both the eGFR ratio and difference in a large cohort of older adults [15]. In addition, CMI was compared with three other creatinine- and cystatin C–based indices in a Taiwanese cohort of 1,141 patients with non-dialysis CKD, in which CMI was significantly associated with mortality and demonstrated superior discrimination as assessed by the C-index [49]. In a large, multicentre study, we have confirmed and extended these prior observations by demonstrating robust associations of CMI with both muscle function and mortality in individuals with non-dialysis CKD, and by highlighting, through direct comparisons, the superiority of CMI over other creatinine/cystatin C-based eGFR metrics in predicting mortality and sarcopenia. Moreover, when CMI was added to age and BMI, this led to an incremental improvement in discrimination for sarcopenia, highlighting its potential to identify at risk individuals.

Our study suggests that simple identification of sarcopenia should be routinely undertaken in people with CKD, not only because of its association with adverse outcomes but also due to the availability of interventions that can reverse sarcopenia in people with CKD. Combined resistance training and protein supplementation have been shown to improve muscle growth and function in frail populations [50,51], and progressive resistance exercise training has demonstrated increased muscle volume in those receiving haemodialysis [52] and non-dialysis CKD [53].

NURTuRE is a large prospective, multicentre study of all-cause CKD with minimal exclusion criteria. It is the largest study to date to validate the relationship between CMI and measures of muscle function in a population with CKD and to assess their relationship with all-cause mortality. Centralised measurement of creatinine and cystatin C minimised potential measurement errors.

As NURTuRE was recruited from UK secondary care centres, the ethnic mix may not represent that of other geographical regions. The results may not apply to differing healthcare systems or primary care settings. Therefore, CMI as a marker of sarcopenia and all-cause mortality should be validated in other global CKD cohorts to confirm its generalisability. We did not assess muscle mass directly using bioelectrical impedance analysis or Dual-Energy X-Ray Absorptiometry and were therefore unable to fully confirm the diagnosis of sarcopenia, hence the use of the term ‘probable sarcopenia’. Given the observational nature of this study, and despite adjustment for multiple confounders, causal inference cannot be established. Because cystatin C levels are higher in individuals with greater adiposity, residual confounding by body fat composition may have affected the association between CMI and outcomes; however, associations persisted in subgroups of BMI. We did not have a measure of GFR that was not derived from serum creatinine or cystatin C concentration and, therefore, could not include eGFR in the multivariable models. A previous study used GFR estimated from β2 microglobulin and found that its inclusion did not remove the association between lower CMI and higher all-cause mortality [15]. Finally, single imputation was used for missing covariate data including UACR, which may underestimate uncertainty.

We have observed that in people with CKD, CMI, a product of eGFRcys and serum creatinine concentration reflective of creatinine generation, was associated with muscle function and all-cause mortality in adjusted models. While previous concerns have been raised about the accuracy of creatinine/cystatin C–based indices at lower GFR, our findings support the validity of CMI in this context. Given its ease of measurement and strong associations with clinically meaningful outcomes, CMI warrants further investigation as a tool to enhance risk stratification and identify individuals at the highest risk of sarcopenia-related complications so that targeted interventions can be undertaken.

## Supporting information

S1 Table Number (%) of missing values of covariates used in regression modelling. Body mass index (BMI), C-reactive protein (CRP), and albumin to creatinine ratio (ACR). Smoking status is defined as yes if the individual is a current or ex-smoker, no if they have never smoked.
(DOCX)

S2 Table Descriptive statistics of Creatinine Muscle Index (CMI) by sex. CMI is measured in mg/day per 1.73 m^2^; SD (standard deviation), IQR refers to interquartile range, Q1 is the first quartile, and Q3 is the third quartile.
(DOCX)

S3 Table Association of log-transformed Creatinine Muscle Index (CMI; per SD increase) with probable sarcopenia, defined by low grip strength. Odds ratios (ORs) were estimated using logistic regression to assess the association between CMI and sarcopenia defined by low grip strength at baseline. Low grip strength was defined as <27 kg for males and <16 kg for females, consistent with the European Working Group on Sarcopenia in Older People 2 (EWGSOP2) definition of probable sarcopenia. ORs are reported per standard deviation increase in log-transformed CMI. Adjustments are for age, white ethnicity, body mass index, smoking status, Charlson Comorbidity Index, urinary albumin-to-creatinine ratio (uACR), and C-reactive protein (CRP).
(DOCX)

S4 Table Association of Creatinine Muscle Index (CMI; per 100 mg/day increase) with probable sarcopenia, defined by low grip strength in subgroups. Low grip strength was defined as <27 kg for males and <16 kg for females, consistent with the European Working Group on Sarcopenia in Older People 2 (EWGSOP2) definition of probable sarcopenia. Odds ratios (ORs) were estimated using logistic regression to assess the association between CMI and sarcopenia defined by low grip strength at baseline. ORs are reported per 100 mg/day per 1.73 m^2^ increase in CMI. Adjustments are for age, white ethnicity, body mass index, smoking status, Charlson Comorbidity Index, urinary albumin-to-creatinine ratio (uACR), and C-reactive protein (CRP).
(DOCX)

S5 Table Associations of log-transformed Creatinine Muscle Index (per SD increase) with All-Cause Mortality. Hazard ratios (HRs) were estimated using Cox proportional hazards regression to assess the association between CMI and all-cause mortality prior to the initiation of kidney replacement therapy (KRT), defined as dialysis or kidney transplantation. HRs are reported per 100 mg/day per 1.73 m^2^ increase in CMI. Adjustments are for age, white ethnicity, body mass index, smoking status, Charlson Comorbidity Index, urinary albumin-to-creatinine ratio (uACR), and C-reactive protein (CRP).
(DOCX)

S6 Table Association of Creatinine Muscle Index (per 100 mg/day increase) with all-cause mortality in subgroups. Hazard ratios (HRs) were estimated using Cox proportional hazards regression to assess the association between CMI and all-cause mortality prior to the initiation of kidney replacement therapy, defined as dialysis or kidney transplantation. HRs are reported per 100 mg/day per 1.73 m^2^ increase in CMI. Adjustments are for age, white ethnicity, body mass index, smoking status, Charlson Comorbidity Index, urinary albumin-to-creatinine ratio, and C-reactive protein.
(DOCX)

S7 Table Partners in the National Unified Renal Translational Research Enterprise, represented on the Joint Steering Committee. (DOCX)

S1 Fig Study participant flow diagram. HGS (hand grip strength), measured in kg, up-and-go (timed up-and-go test) measured in seconds, The EQ-5D-3L measures quality of life as a standardised health-related quality-of-life measure.
(PDF)

S2 Fig Kernel density plot for Creatinine Muscle Index in males and females. (PDF)

S3 Fig Association of Creatinine Muscle Index with the log relative hazard of death before kidney replacement therapy, stratified by sex using restricted cubic splines with 3 knots, unadjusted. The solid lines represent the spline fits for males (blue) and females (red), with shaded areas indicating the 95% confidence intervals. In females, increasing CMI from 726.6 to 1020.3 mg/day/1.73 m^2^ was associated with a lower risk of death (HR = 0.34, 95% CI 0.28–0.40). In males, increasing CMI from 586 to 841 mg/day/1.73 m^2^ was associated with a lower risk of death (HR = 0.23, 95% CI 0.17–0.32).
(PDF)

S4 Fig Association of Creatinine Muscle Index with the log relative hazard of death before kidney replacement therapy, stratified by sex using restricted cubic splines with 3 knots, adjusted for age, white ethnicity, body mass index, smoking, Charlson Comorbidity Index, albumin-to-creatinine ratio, and C-reactive protein. The solid lines represent the spline fits for males (blue) and females (red), with shaded areas indicating the 95% confidence intervals. In males, increasing CMI from 726.6 to 1020.3 mg/day/1.73 m^2^ was associated with a lower risk of death (HR = 0.60, 95% CI 0.49–0.73). In females, increasing CMI from 586 to 841 mg/day/1.73 m^2^ corresponded to a hazard ratio of 0.50 (95% CI 0.36–0.71).
(PDF)

S1 File NURTuRE-CKD study protocol. (PDF)

S1 Checklist STROBE checklist. Checklist of items that should be included in reports of cohort studies. This checklist is reproduced from the STROBE Statement (Strengthening the Reporting of Observational Studies in Epidemiology) and is licensed under the Creative Commons Attribution 4.0 International (CC BY 4.0). Source: https://www.strobe-statement.org/.
(DOCX)

## Abbreviations

AUC: area under the curve
BMI: body mass index
CCI: Charlson Comorbidity Index
CI: confidence interval
CKD: chronic kidney disease
CKD-EPI: Chronic Kidney Disease Epidemiology Collaboration
CMI: Creatinine Muscle Index
CRP: C-Reactive Protein
EWGSOP: European Working Group on Sarcopenia in Older People
GFR: glomerular filtration rate
HR: hazard ratio
HRQoL: health-related quality-of-life
KPS: Karnofsky Performance Score
KRT: kidney replacement therapy
NURTuRE: National Unified Renal Translational Research Enterprise
SD: standard deviation
TUG: timed get-up-and-go
uACR: urine albumin-to-creatinine ratio
