# Supplementary material for: Associations of Creatinine Muscle Index with markers of sarcopenia and mortality in chronic kidney disease: A prospective cohort study
Source: PLoS Med. 2026 Feb 12;23(2):e1004775. doi: 10.1371/journal.pmed.1004775 (PMC12900331; doi:10.1371/journal.pmed.1004775)
Supplement: S1 Table — Body mass index (BMI), C-reactive protein (CRP), and albumin to creatinine ratio (ACR). Smoking status is defined as yes if the individual is a current or ex-smoker, no if they have never smoked. (DOCX) [file pmed.1004775.s001.docx]

**S1 Table -** Number (%) of missing values of covariates used in regression modelling

| **Variable** | **Number (%) missing**  **Male (n = 1723)** | **Number (%) missing  Female (n = 1207)** |
| --- | --- | --- |
| BMI | 32 (1.9%) | 44 (3.6%) |
| Smoking status | 19 (1.1%) | 19 (1.6%) |
| CRP | 1 (0.1%) | 2 (0.2%) |
| UACR | 128 (7.4%) | 119 (9.9%) |

Body mass index (BMI), C-reactive protein (CRP), urine albumin to creatinine ratio (UACR). Smoking status is defined as yes if the individual is a current or ex-smoker, no if they have never smoked.
