# Supplementary material for: Associations of Creatinine Muscle Index with markers of sarcopenia and mortality in chronic kidney disease: A prospective cohort study
Source: PLoS Med. 2026 Feb 12;23(2):e1004775. doi: 10.1371/journal.pmed.1004775 (PMC12900331; doi:10.1371/journal.pmed.1004775)
Supplement: S2 Table — CMI is measured in mg/day per 1.73 m2; SD (standard deviation), IQR refers to interquartile range, Q1 is the first quartile, and Q3 is the third quartile. (DOCX) [file pmed.1004775.s002.docx]

**S2 Table** - Descriptive Statistics of Creatinine Muscle Index by Sex

| count | mean | median | SD | IQR | minimum | maximum | Q1 | Q3 |
| --- | --- | --- | --- | --- | --- | --- | --- | --- |
| Total  2930 | 826 | 795 | 235 | 297 | 286 | 2214 | 661 | 958 |
| Male  1723 | 892 | 864 | 232 | 294 | 286 | 2214 | 727 | 1020 |
| Female  1207 | 730 | 704 | 204 | 255 | 329 | 1664 | 586 | 841 |

*Creatinine Muscle Index (CMI) is measured in mg/day per 1.73 m²; SD (standard deviation), IQR refers to interquartile range, Q1 is the first quartile, and Q3 is the third quartile.*
