# Supplementary material for: Associations of Creatinine Muscle Index with markers of sarcopenia and mortality in chronic kidney disease: A prospective cohort study
Source: PLoS Med. 2026 Feb 12;23(2):e1004775. doi: 10.1371/journal.pmed.1004775 (PMC12900331; doi:10.1371/journal.pmed.1004775)
Supplement: S3 Table — Odds ratios (ORs) were estimated using logistic regression to assess the association between CMI and sarcopenia defined by low grip strength at baseline. Low grip strength was defined as <27 kg for males and <16 kg for females, consistent with the European Working Group on Sarcopenia in Older People 2 (EWGSOP2) definition of probable sarcopenia. ORs are reported per standard deviation increase in log-transformed CMI. Adjustments are for age, white ethnicity, body mass index, smoking status, Charlson Comorbidity Index, urinary albumin-to-creatinine ratio (uACR), and C-reactive protein (CRP). (DOCX) [file pmed.1004775.s003.docx]

**S3 Table –** Association of log-transformed creatinine muscle index (per SD increase) with probable sarcopenia, defined by low grip strength

|  | **Unadjusted** | | **Adjusted** | |
| --- | --- | --- | --- | --- |
|  | OR (95% CI) | P value | OR (95% CI) | P value |
| **Male** | 0.38 (0.34,0.44) | < 0.001 | 0.51 (0.43,0.59) | < 0.001 |
| **Female** | 0.46 (0.4,0.54) | < 0.001 | 0.69 (0.58,0.83) | < 0.001 |

*Odds ratios (ORs) were estimated using logistic regression to assess the association between creatinine muscle index (CM) and sarcopenia defined by low grip strength at baseline. Low grip strength was defined as <27 kg for males and <16 kg for females, consistent with the European Working Group on Sarcopenia in Older People 2 (EWGSOP2) definition of probable sarcopenia. ORs are reported per standard deviation increase in log-transformed creatinine muscle index. Adjustments are for age, white ethnicity, body mass index, smoking status, Charlson Comorbidity Index, urinary albumin-to-creatinine ratio (uACR) and C-reactive protein (CRP).*
