# Supplementary material for: Associations of Creatinine Muscle Index with markers of sarcopenia and mortality in chronic kidney disease: A prospective cohort study
Source: PLoS Med. 2026 Feb 12;23(2):e1004775. doi: 10.1371/journal.pmed.1004775 (PMC12900331; doi:10.1371/journal.pmed.1004775)
Supplement: S5 Table — Hazard ratios (HRs) were estimated using Cox proportional hazards regression to assess the association between CMI and all-cause mortality prior to the initiation of kidney replacement therapy (KRT), defined as dialysis or kidney transplantation. HRs are reported per 100 mg/day per 1.73 m2 increase in CMI. Adjustments are for age, white ethnicity, body mass index, smoking status, Charlson Comorbidity Index, urinary albumin-to-creatinine ratio (uACR), and C-reactive protein (CRP). (DOCX) [file pmed.1004775.s005.docx]

**S5 Table–** Associations of log-transformed creatinine muscle index (per SD increase) with All-Cause Mortality

|  | **Unadjusted** | | **Adjusted** | |
| --- | --- | --- | --- | --- |
|  | HR (95% CI) | P value | HR (95% CI) | P value |
| **Male** | 0.47 (0.42, 0.52) | < 0.001 | 0.69 (0.60, 0.78) | < 0.001 |
| **Female** | 0.37 (0.32, 0.44) | < 0.001 | 0.64 (0.52, 0.78) | < 0.001 |

*Hazard ratios (HRs) were estimated using Cox proportional hazards regression to assess the association between CMI and all-cause mortality prior to the initiation of kidney replacement therapy (KRT), defined as dialysis or kidney transplantation. HRs are reported per 100 mg/day per 1.73 m² increase in CMI. Adjustments are for age, white ethnicity, body mass index, smoking status, Charlson Comorbidity Index, urinary albumin-to-creatinine ratio (uACR) and C-reactive protein (CRP).*
