# Supplementary material for: Associations of Creatinine Muscle Index with markers of sarcopenia and mortality in chronic kidney disease: A prospective cohort study
Source: PLoS Med. 2026 Feb 12;23(2):e1004775. doi: 10.1371/journal.pmed.1004775 (PMC12900331; doi:10.1371/journal.pmed.1004775)
Supplement: S6 Table — Hazard ratios (HRs) were estimated using Cox proportional hazards regression to assess the association between CMI and all-cause mortality prior to the initiation of kidney replacement therapy, defined as dialysis or kidney transplantation. HRs are reported per 100 mg/day per 1.73 m2 increase in CMI. Adjustments are for age, white ethnicity, body mass index, smoking status, Charlson Comorbidity Index, urinary albumin-to-creatinine ratio, and C-reactive protein. (DOCX) [file pmed.1004775.s006.docx]

**S6 Table -** Association of creatinine muscle index (per 100 mg/day increase) with all-cause mortality in subgroups

| Male | | | | | | |
| --- | --- | --- | --- | --- | --- | --- |
|  | Unadjusted | | | Adjusted | | |
|  | HR | 95% CI | P value | HR | 95% CI | P value |
| BMI < 30 | 0.66 | 0.61, 0.72 | <0.001 | 0.83 | 0.75, 0.92 | <0.001 |
| BMI ≥ 30 | 0.71 | 0.65, 0.77 | <0.001 | 0.83 | 0.75, 0.92 | <0.001 |
| Age < 65 | 0.7 | 0.61, 0.79 | <0.001 | 0.82 | 0.71, 0.94 | 0.006 |
| Age ≥ 65 | 0.74 | 0.68, 0.80 | <0.001 | 0.84 | 0.77, 0.91 | <0.001 |
| Female | | | | | | |
|  | Unadjusted | | | Adjusted | | |
|  | HR | 95% CI | P value | HR | 95% CI | P value |
| BMI < 30 | 0.56 | 0.48, 0.65 | <0.001 | 0.78 | 0.65, 0.93 | 0.005 |
| BMI ≥ 30 | 0.52 | 0.44, 0.62 | <0.001 | 0.75 | 0.62, 0.91 | 0.004 |
| Age < 65 | 0.58 | 0.45, 0.74 | <0.001 | 0.76 | 0.57, 1.01 | 0.056 |
| Age ≥ 65 | 0.62 | 0.55, 0.71 | <0.001 | 0.78 | 0.67, 0.91 | 0.001 |

*Hazard ratios (HRs) were estimated using Cox proportional hazards regression to assess the association between CMI and all-cause mortality prior to the initiation of kidney replacement therapy, defined as dialysis or kidney transplantation. HRs are reported per 100 mg/day per 1.73 m² increase in CMI. Adjustments are for age, white ethnicity, body mass index (BMI), smoking status, Charlson Comorbidity Index, urinary albumin-to-creatinine ratio and C-reactive protein. .*
