# Supplementary material for: Associations of Creatinine Muscle Index with markers of sarcopenia and mortality in chronic kidney disease: A prospective cohort study
Source: PLoS Med. 2026 Feb 12;23(2):e1004775. doi: 10.1371/journal.pmed.1004775 (PMC12900331; doi:10.1371/journal.pmed.1004775)
Supplement: S7 Table — (DOCX) [file pmed.1004775.s007.docx]

**S7 Table.** Partners in the National Unified Renal Translational Research Enterprise, represented on the Joint Steering Committee

| **Name** | **Contribution** |
| --- | --- |
| Kidney Research UK | Coordination, management of funding,  convene and chair Joint Steering Committee, convene Strategic Oversite and Access Committee |
| Academic steering group | Study design, conduct of study, analysis and interpretation of data, publications |
| UK Renal Registry | Collection, management and storage of study data, collection of outcome data, analysis of data |
| NIHR National Biosample Centre | Storage and curation of liquid biosamples |
| Human Biomaterials Resource Centre, University of Birmingham | Storage and curation of kidney biopsy slides and tissue, digital scanning of biopsy slides, multiplex immunostaining |
| University of Bristol | Project management |
| Hopitaux Universitaires Geneve | Biomarker analysis on liquid biosamples |
| University of Nottingham | Study sponsor, chief investigator, project management |
| Patient representatives | Provide patient perspective, advise on participant facing documentation |
| UCB Biopharma | Funding, scientific advice, biosample analysis |
| Evotec International GmbH | Funding, scientific advice, biosample analysis |
| AstraZeneca | Funding, scientific advice, biosample analysis |
| AbbVie | Funding, scientific advice |
| Travere Therapeutics | Funding, scientific advice |
