# Supplementary figures and images for: Associations of Creatinine Muscle Index with markers of sarcopenia and mortality in chronic kidney disease: A prospective cohort study
Source: PLoS Med. 2026 Feb 12;23(2):e1004775. doi: 10.1371/journal.pmed.1004775 (PMC12900331; doi:10.1371/journal.pmed.1004775)

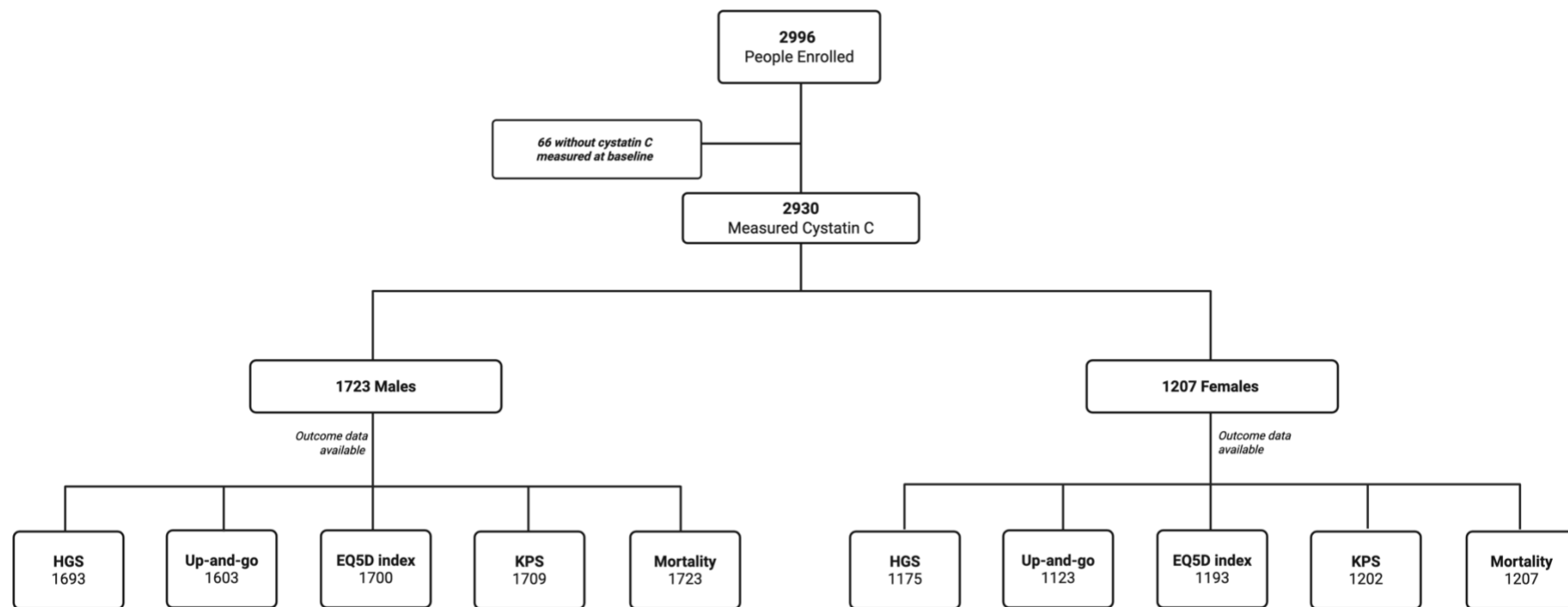

Supplement: S1 Fig — HGS (hand grip strength), measured in kg, up-and-go (timed up-and-go test) measured in seconds, The EQ-5D-3L measures quality of life as a standardised health-related quality-of-life measure. (PDF) [file pmed.1004775.s008.pdf]

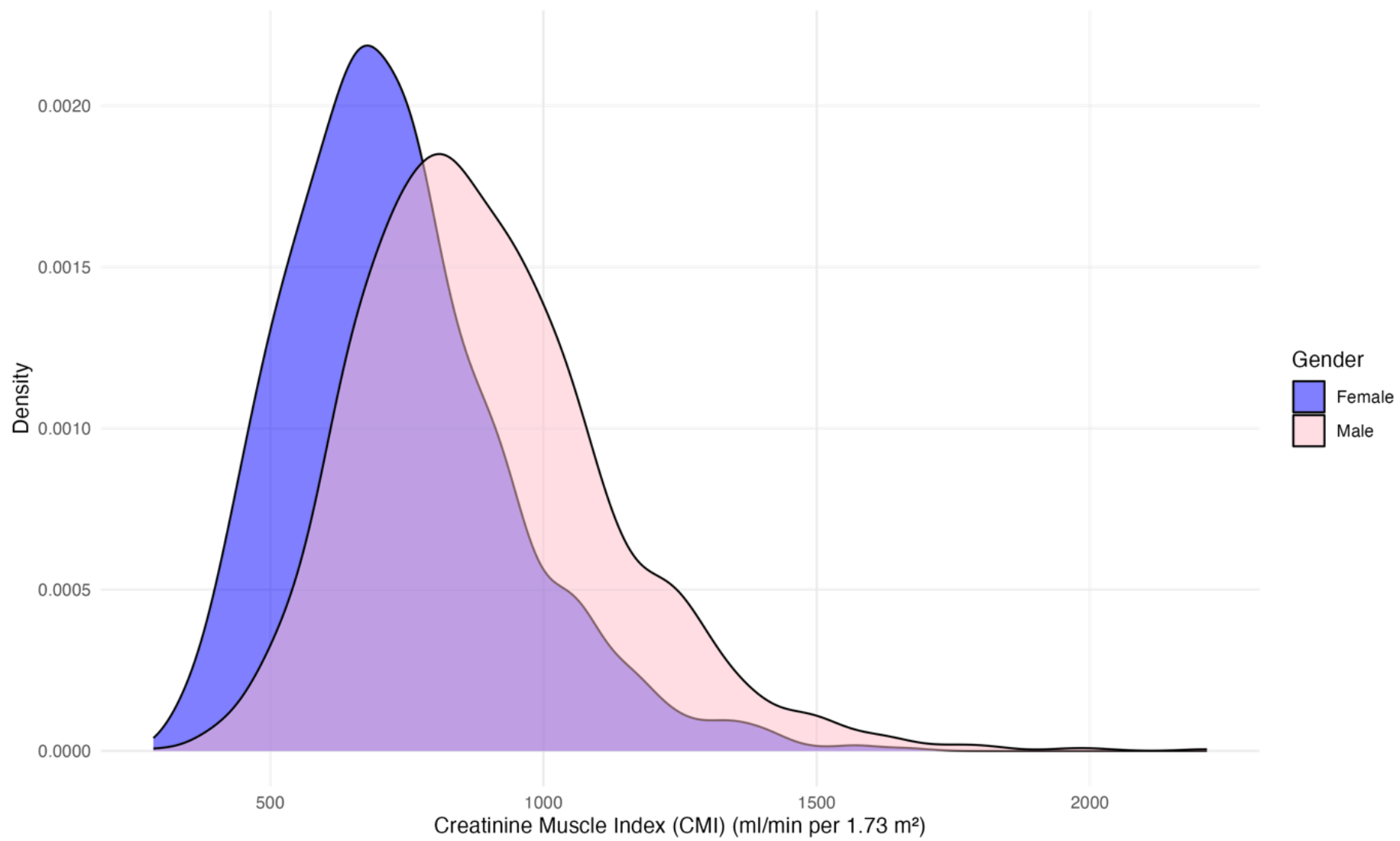

Supplement: S2 Fig — (PDF) [file pmed.1004775.s009.pdf]

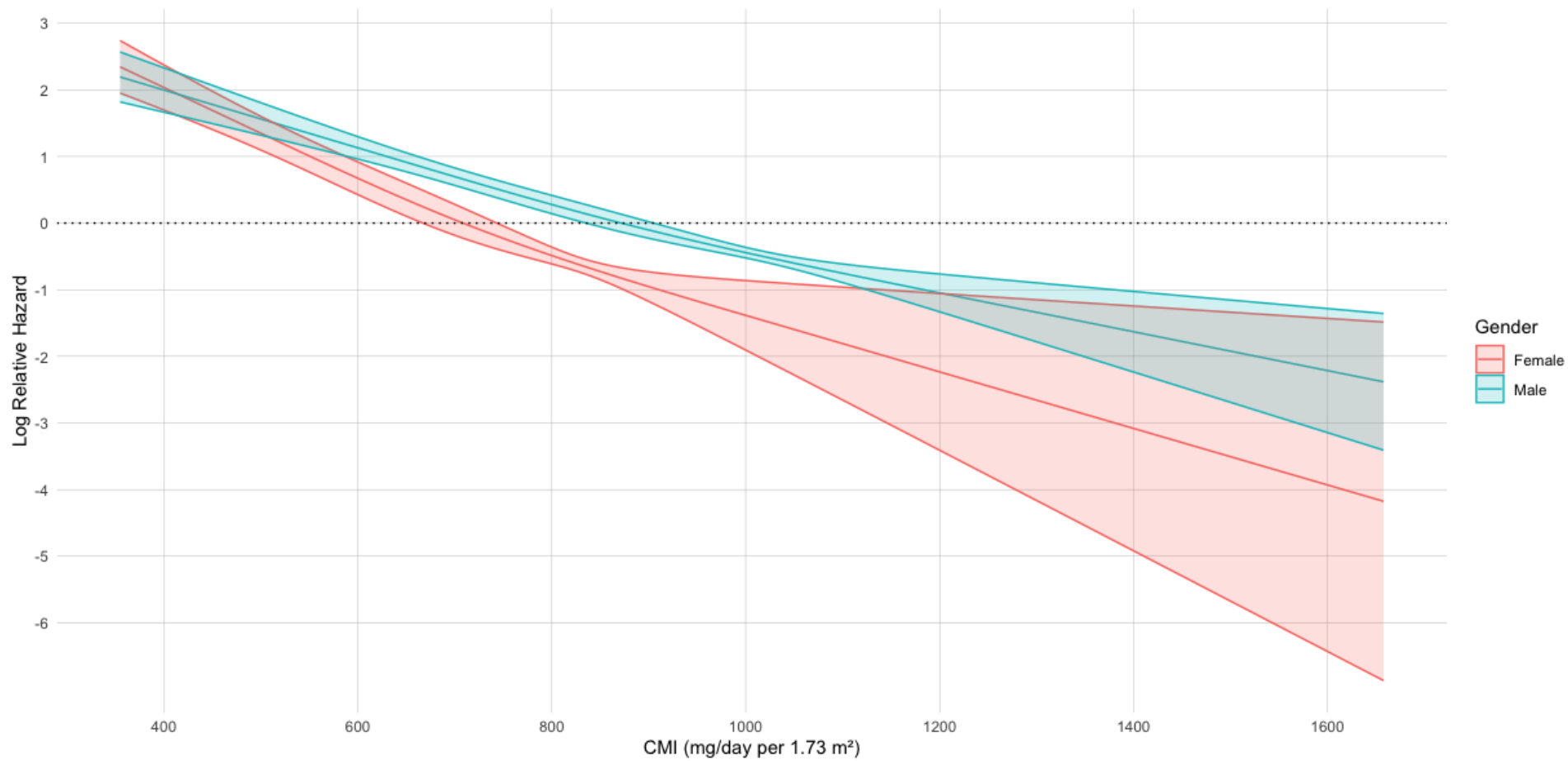

Supplement: S3 Fig — The solid lines represent the spline fits for males (blue) and females (red), with shaded areas indicating the 95% confidence intervals. In females, increasing CMI from 726.6 to 1020.3 mg/day/1.73 m2 was associated with a lower risk of death (HR = 0.34, 95% CI 0.28–0.40). In males, increasing CMI from 586 to 841 mg/day/1.73 m2 was associated with a lower risk of death (HR = 0.23, 95% CI 0.17–0.32). (PDF) [file pmed.1004775.s010.pdf]

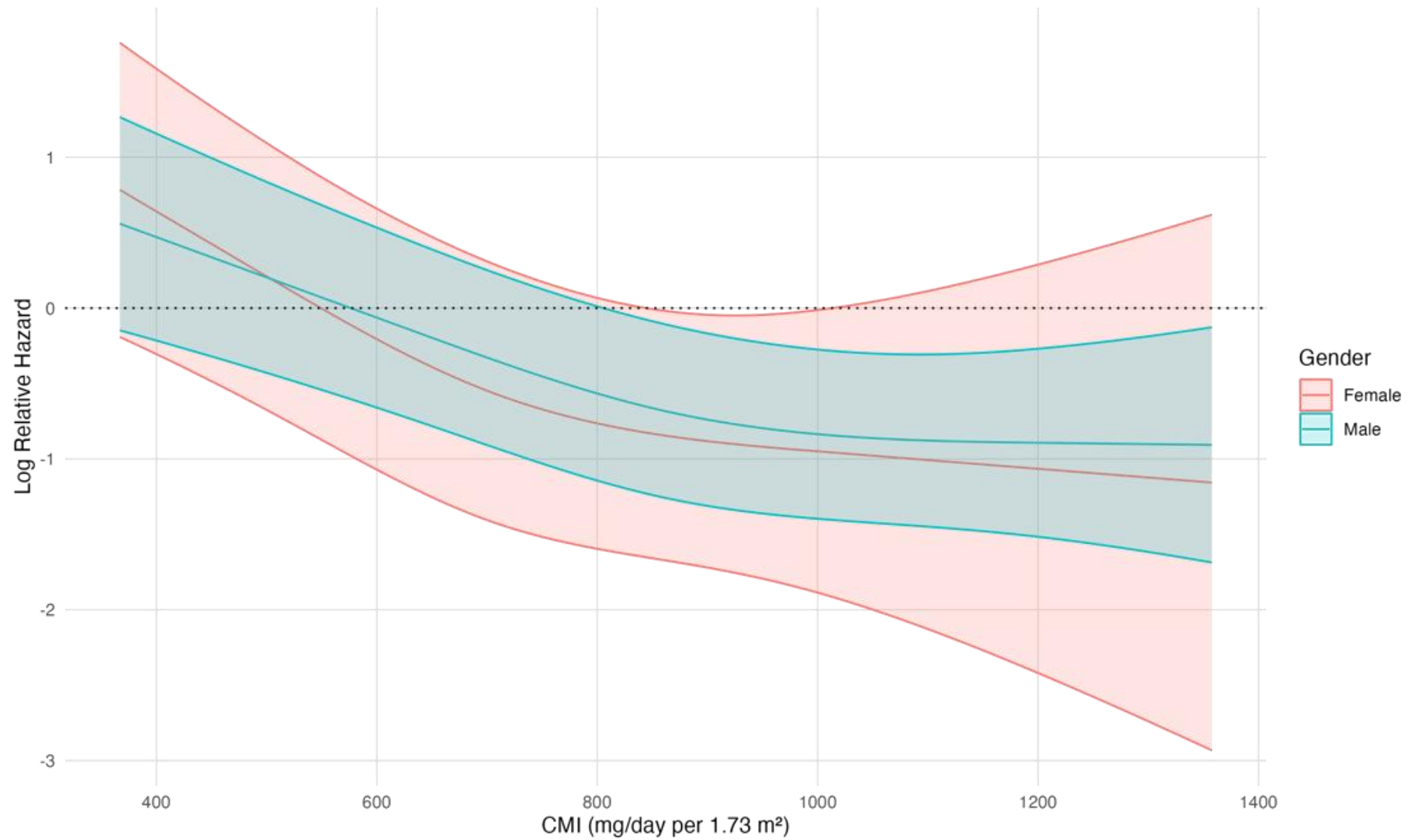

Supplement: S4 Fig — The solid lines represent the spline fits for males (blue) and females (red), with shaded areas indicating the 95% confidence intervals. In males, increasing CMI from 726.6 to 1020.3 mg/day/1.73 m2 was associated with a lower risk of death (HR = 0.60, 95% CI 0.49–0.73). In females, increasing CMI from 586 to 841 mg/day/1.73 m2 corresponded to a hazard ratio of 0.50 (95% CI 0.36–0.71). (PDF) [file pmed.1004775.s011.pdf]
