# Supplementary material for: Associations of Creatinine Muscle Index with markers of sarcopenia and mortality in chronic kidney disease: A prospective cohort study
Source: PLoS Med. 2026 Feb 12;23(2):e1004775. doi: 10.1371/journal.pmed.1004775 (PMC12900331; doi:10.1371/journal.pmed.1004775)
Supplement: S1 File — (PDF) [file pmed.1004775.s012.pdf]

NATIONAL UNIFIED RENAL TRANSLATIONAL RESEARCH ENTERPRISE – CHRONIC KIDNEY DISEASE

Version 4.4 11/4/2024

Short title: **NURTuRE - CKD**

Acronym: **NURTuRE - CKD**

Trial Registration: www.clinicaltrials.gov

ISRCTN:

IRAS Project ID: 211479

Trial Sponsor: University of Nottingham

Sponsor reference: 16091

Funding Source: Kidney Research UK

This protocol is confidential and the property of the University of Nottingham. No part of it may be transmitted, reproduced, published, or used by other persons without prior written authorisation from the University of Nottingham

TRIAL / STUDY PERSONNEL AND CONTACT DETAILS

Sponsor: University of Nottingham
Contact name: Mr Ali Alshukry
Research and Innovation
University of Nottingham
E-Floor, Yang Fujia Building
Jubilee Campus
Nottingham
NG8 1BB
Email: sponsor@nottingham.ac.uk

Chief investigator: Maarten Taal
Professor of Medicine
Phone: 01332 724618
Email: m.taal@nottingham.ac.uk

Co-investigators: Paul Cockwell
Consultant Nephrologist
Email: paul.cockwell@uhb.nhs.uk

Philip Kalra
Consultant Nephrologist
Email: philip.kalra@srft.nhs.uk

Simon Fraser
Associate Professor of Public Health
Email: S.Fraser@soton.ac.uk

David Wheeler
Professor of Kidney Medicine
Email: d.wheeler@ucl.ac.uk

Trial / Study Statistician: Dr Retha Steenkamp Head of Operations
UK Renal Registry
Email: Retha.steenkamp@renalregistry.nhs.uk

Trial / Study Coordinating Centre:

Maarten Taal
Centre for Kidney Research and Innovation
Division of Medical Sciences and Graduate Entry Medicine, University of Nottingham
Email: m.taal@nottingham.ac.uk

This protocol is confidential and the property of the University of Nottingham. No part of it may be transmitted, reproduced, published, or used by other persons without prior written authorisation from the University of Nottingham

SYNOPSIS

Title	NATIONAL UNIFIED RENAL TRANSLATIONAL RESEARCH ENTERPRISE – CHRONIC KIDNEY DISEASE
Acronym	NURTuRE - CKD
Short title	NURTuRE - CKD
Chief Investigator	Maarten Taal
Objectives	To investigate determinants of and risk factors for clinically important adverse outcomes in people with chronic kidney disease.
Trial Configuration	Multicentre prospective cohort study
Setting	Secondary care
Sample size estimate	For 3000 participants, an event rate for death of 5/100 participant years, an ESKD rate of 7-10/100 participant years and up to 5.5 years follow-up, the study will have 80% power at two-sided 5% significance level to detect a minimum HR of 1.52-1.70 for death and 1.39-1.47 for ESKD associated with a biomarker with baseline frequency of 50%.
Number of participants	3000 participants with CKD<br>100 participants without CKD (normal controls for biomarker assays)
Eligibility criteria	Inclusion criteria<br>1. Over 18 years of age<br>2. Estimated GFR 59-15ml/min/1.73m² or eGFR >60ml/min/1.73m² and urine albumin to creatinine ratio >30mg/mmol 3. Seen at least once in a Nephrology Clinic<br>4. Willing to participate in two study visits<br>5. Able to give informed consent and participate in study procedures<br><br>Exclusion criteria<br>1. Recipient of a solid organ transplant<br>2. Expected survival less than 1 year<br>3. Acute kidney injury within 3 months of recruitment<br>4. Major Acute Cardiovascular Event (MACE) within 3 months of recruitment<br>5. Primary Focal and Segmental Glomerulosclerosis and Idiopathic Minimal Change Disease<br>6. Current or planned chemotherapy for cancer
Description of interventions	Participants with CKD who meet the eligibility criteria and give informed consent will be asked to attend a study visit where they will be asked to complete a number of quality of life and function status assessments, anthropomorphic assessments and blood and urine tests. The same

This protocol is confidential and the property of the University of Nottingham. No part of it may be transmitted, reproduced, published, or used by other persons without prior written authorisation from the University of Nottingham

	assessments will be repeated at a second study visit, 12-66 months after the first visit. Should participants consent they will be asked to complete an annual questionnaire by post. Participants without CKD will attend one study visit only where anthropomorphic assessment and blood and urine test will occur.
Duration of study	Up to 15 years
Randomisation and blinding	Not applicable
Outcome measures	1. Progression of CKD to kidney failure as defined a reduction in estimated GFR to <15ml/min/1.73m^2 or initiation of kidney replacement therapy<br>2. Major acute cardiovascular events
Statistical methods	The main analyses will be multivariable looking at the predictive performance of biomarkers alone and in combination with other socio-demographic and clinical variables. Time-to-event analyses (KaplanMeier, Cox regression) will be used to assess the relationship between baseline/follow-up variables and end points such as all-cause mortality, cardiovascular and ESKD assuming that the proportionality assumption is met.

This protocol is confidential and the property of the University of Nottingham. No part of it may be transmitted, reproduced, published, or used by other persons without prior written authorisation from the University of Nottingham

ABBREVIATIONS

AE Adverse Event

AKI Acute Kidney Injury

CKD Chronic Kidney Disease

CI Chief Investigator overall

CRF Case Report Form

CVE Cardiovascular Events

DMC Data Monitoring Committee

eGFR estimated Glomerular Filtration Rate

ESKD End-stage Kidney Disease

FSGS Focal segmental glomerulosclerosis

GCP Good Clinical Practice

GFR Glomerular Filtration Rate

ICF Informed Consent Form

MACE Major Acute Cardiovascular Events

NHS National Health Service

PI Principal Investigator at a local centre

PIS Participant Information Sheet

REC Research Ethics Committee

R&D Research and Development department

SAE Serious Adverse Event

This protocol is confidential and the property of the University of Nottingham. No part of it may be transmitted, reproduced, published, or used by other persons without prior written authorisation from the University of Nottingham

TABLE OF CONTENTS

SYNOPSIS **3**
ABBREVIATIONS **5**
TRIAL / STUDYBACKGROUND INFORMATION AND RATIONALE **8**
TRIAL / STUDY OBJECTIVES AND PURPOSE **9**
PURPOSE 9
PRIMARY OBJECTIVE 9
SECONDARY OBJECTIVES 9
DETAILS OF PRODUCT(S) **9**
TRIAL / STUDY DESIGN **9**
TRIAL / STUDY CONFIGURATION 9
Prospective, multicentre cohort study 9
Primary endpoints 9
Secondary endpoints 9
Stopping rules and discontinuation 10
RANDOMIZATION AND BLINDING 10
TRIAL/STUDY MANAGEMENT 10
DURATION OF THE TRIAL / STUDY AND PARTICIPANT INVOLVEMENT 11
End of the Trial 11
SELECTION AND WITHDRAWAL OF PARTICIPANTS 11
Recruitment 11
Eligibility criteria: Participants with CKD 12
Inclusion criteria 12
Exclusion criteria 12
Eligibility criteria: Participants without CKD (controls) 12
Inclusion criteria 12
Exclusion criteria 12
Expected duration of participant participation 13
Removal of participants from therapy or assessments/Participant Withdrawal 13
Informed consent 13
STUDY REGIMEN **13**
Participants with CKD: Study visit 1: 13
Participants acting as controls: Study Visit 1: 16
Participants with CKD: Study Visit 2 16
Compliance 16
Criteria for terminating trial 17
RADIATION EXPOSURE 17
TRANSPORT AND STORAGE OF THE TISSUES 17
LABORATORY ANALYSES 17
STATISTICS **18**
Methods 18
Sample size and justification 18

This protocol is confidential and the property of the University of Nottingham. No part of it may be transmitted, reproduced, published, or used by other persons without prior written authorisation from the University of Nottingham

ADVERSE EVENTS **20**
ETHICAL AND REGULATORY ASPECTS **20**
ETHICS COMMITTEE AND REGULATORY APPROVALS 20
INFORMED CONSENT AND PARTICIPANT INFORMATION 20
RECORDS 21
Case Report Forms 21
Sample Labelling 21
Source documents 21
Direct access to source data / documents 21
DATA PROTECTION 22
QUALITY ASSURANCE & AUDIT **22**
INSURANCE AND INDEMNITY 22
TRIAL CONDUCT 22
TRIAL DATA 22
RECORD RETENTION AND ARCHIVING 23
DISCONTINUATION OF THE TRIAL BY THE SPONSOR 23
STATEMENT OF CONFIDENTIALITY 23
PUBLICATION AND DISSEMINATION POLICY **23**
USER AND PUBLIC INVOLVEMENT **24**
STUDY FINANCES **24**
Funding source 24
Participant stipends and payments 24
SIGNATURE PAGES **25**
REFERENCES **27**

This protocol is confidential and the property of the University of Nottingham. No part of it may be transmitted, reproduced, published, or used by other persons without prior written authorisation from the University of Nottingham

TRIAL / STUDYBACKGROUND INFORMATION AND RATIONALE

Chronic kidney disease (CKD) affects approximately 10% of the adult population worldwide [1] and is associated with increased risk of multiple adverse outcomes including end-stage kidney disease (ESKD), all-cause mortality, cardiovascular events (CVE) and acute kidney injury (AKI) [2-4]. The risk profile associated with CKD is however heterogeneous with the majority being at relatively low risk. There is therefore a need for methods to better predict adverse clinical outcomes in individuals with CKD, so that interventions to reduce risk can be targeted to those most likely to benefit, whereas those at low risk can be spared unnecessary intervention and anxiety. A risk prediction equation has been developed to estimate the risk of progression to ESKD using simple demographic and biochemical variables (age, gender, estimated glomerular filtration rate, urine albumin to creatinine ratio, serum albumin, bicarbonate, calcium and phosphate) [5, 6], but there is currently no validated method to assess the likelihood of future cardiovascular events or other adverse outcomes associated with CKD.

Most forms of CKD progress slowly over years or decades before reaching ESKD. At present the only primary outcomes accepted by drug regulatory authorities for trials of renoprotective drugs in CKD are ESKD and ESKD or doubling of serum creatinine. This has proved to be a major barrier to the development of novel renoprotective drugs because clinical trials based on these primary outcomes require large numbers of participants and prolonged follow-up to accrue sufficient events. As a result, trials of new renoprotective drugs are extremely costly and this is likely to have contributed to a lack of new therapies for CKD. Identifying novel biomarkers that reflect the activity of biological pathways known to contribute to CKD progression and the associated adverse outcomes is likely to facilitate the development of surrogate outcome measures that would give drug developers greater confidence of a therapeutic effect prior to embarking on large clinical trials. This strategy may also lead to the acceptance of new primary outcome measures for trials [7]. Together these developments will be expected to substantially lower the cost of testing new renoprotective drugs and thereby facilitate the development of novel therapies.

The NURTuRE-CKD study aims to develop and validate robust risk prediction methods for adverse outcomes associated with CKD using a combination of previously identified risk factors and novel biomarkers. A key novel dimension, not included in other cohort studies of CKD, will be analysis of kidney biopsy specimens. This will allow evaluation of biomarkers at the site of pathological damage in the kidneys as well as in blood and urine. Furthermore, storage of serum, plasma, urine, RNA and DNA will allow the investigators to study novel biomarkers that may not yet have been identified in order to ensure maximum benefit from the data collected.

The NURTuRE project is a collaboration between several independent investigators working in partnership with pharmaceutical companies engaged in the development of new treatments for CKD. This collaborative approach is expected to maximise the scientific value of the study and to assist in drug development and testing. Stored biosamples will also be made available to investigators outside the steering committee and to pharmaceutical companies (via an independent access committee) to further increase the value of the dataset for research. As such the NURTuRE project represents an important development in the progress of renal research in the UK that will act as a springboard for similar developments in research on other aspects of kidney disease and specifically fulfils several of the objectives outlined in the recent UK Kidney Research Consortium’s Research Strategy Document.

This protocol is confidential and the property of the University of Nottingham. No part of it may be transmitted, reproduced, published, or used by other persons without prior written authorisation from the University of Nottingham

TRIAL / STUDY OBJECTIVES AND PURPOSE

PURPOSE

To investigate determinants of and risk factors for clinically important adverse outcomes in people with chronic kidney disease.

PRIMARY OBJECTIVE

To investigate determinants of and risk factors for clinically important adverse outcomes in people with chronic kidney disease, including:

- Death
- Major Acute Cardiovascular Events (MACE)
- Progression of CKD (KDIGO definition)
- End-stage kidney disease (ESKD)
- Acute Kidney Injury (AKI)
- Hospital admissions
- Infections requiring hospital admission
- New Cancer diagnosis
- Hip fractures

SECONDARY OBJECTIVES

1. To identify and validate novel biomarkers to predict clinically important outcomes in people with CKD.
2. To investigate novel mechanisms underlying the association between CKD and multiple adverse outcomes.
3. To assess the performance of previously published risk scores for people with CKD (external validation).
4. To develop and internally validate new risk scores to predict adverse outcomes in people with CKD and evaluate the potential benefit of adding novel biomarkers to existing risk scores.
5. To assess the relationship between severity of CKD, comorbid conditions and Quality of Life.
6. To investigate the resource use and costs of patients with CKD at different levels of severity with varying morbidity.

DETAILS OF PRODUCT(S)

Not applicable

TRIAL / STUDY DESIGN

TRIAL / STUDY CONFIGURATION

Prospective, multicentre cohort study

Primary endpoints

1. Progression of chronic kidney disease to kidney failure (defined sustained decrease to <15mL/min/1.73m^2 or initiation of kidney replacement therapy).
2. Major Acute Cardiovascular Event (MACE) – cardiac death, non-fatal myocardial infarction, cerebral infarction or intracerebral haemorrhage, arterial revascularisation

Secondary endpoints

a. Death from any cause

This protocol is confidential and the property of the University of Nottingham. No part of it may be transmitted, reproduced, published, or used by other persons without prior written authorisation from the University of Nottingham

b. Progression of CKD (KDIGO definition)
c. Rate of GFR decline
d. Percentage GFR decline (30% or 40% or 50%)
e. End-stage kidney disease (ESKD)
f. Acute Kidney Injury (AKI)
g. New diagnosis of cardiac failure
h. Unplanned hospital admissions
i. Infections requiring hospital admission
j. Cancer diagnosis
k. Hip fractures

Stopping rules and discontinuation

This is an observational study. Participants will be withdrawn from the study only if they withdraw their consent. The study will be discontinued only if for unforeseen circumstances it becomes clear that it is no longer feasible.

RANDOMIZATION AND BLINDING

Not applicable

TRIAL/STUDY MANAGEMENT

The Chief Investigator has overall responsibility for the study and shall oversee all study management. The study will be managed from the coordinating centre at the University of Nottingham by a project manager supported by the Chief Investigator. A Study Management Committee (SMC) will further support and coordinate delivery of the study and will be comprised of:

- Chief Investigator
- Principle Investigators
- Project Manager
- Data Manager (based at the UK Renal Registry)
- Statistician
- Kidney Research UK representative
- Research Nurses

The SMC will meet regularly (monthly) by teleconference with face to face meetings as required.

A Joint Steering Committee (JSC) will provide oversight of the study and will review progress against the objectives and milestones. The JSC will be comprised of:

- Kidney Research UK Director of Research Operations (JSC Chair)
- Chief Investigator (for the NURTuRE-CKD)
- Representative from each of the three pharmaceutical company partners (initial members)
- Patient Representative
- UK Renal Registry Representative
- UK Biobank Representative
- University of Birmingham (HBRC) Representative
- Geneva University (HUG) Representative
- Chief Investigator (for the NURTuRE-Nephrotic Syndrome study)

Top level oversight of the biobanked samples will be provided by the NURTuRE Strategy Committee comprised of:

This protocol is confidential and the property of the University of Nottingham. No part of it may be transmitted, reproduced, published, or used by other persons without prior written authorisation from the University of Nottingham

- An independent Chair, appointed by Kidney Research UK
- Patient representatives
- One to three Independent Academic representatives

The Strategy Committee will provide governance and oversight of the biorepository resources at Milton Keynes and the University of Birmingham and will appoint and oversee an Independent Biosample Access Committee that will consider and approve applications for access to all specimens in the biorepositories from independent investigators who wish to make use of the samples.

The data custodian will be the Chief Investigator.

DURATION OF THE TRIAL / STUDY AND PARTICIPANT INVOLVEMENT

Study Duration: Enrolment will commence in the **2nd quarter of 2017** and will continue for 12 months or until at least 3000 participants with CKD and 100 participants without CKD (50 with diabetes and 50 without diabetes) have been recruited. Follow-up will continue for up to 15 years. Analysis of data will continue for at least 3 years after the follow-up period. The total duration of the study will therefore be up to 20 years.

Participant Duration: Participants with CKD will be actively involved for up to 66 months only. After the follow-up study visit outcome data will be collected via the UK Renal Registry for a further 14 years. Participants will be sent a questionnaire annually by post to assess quality of life (EQ-5D-5L) and health care resource utilisation during the previous year but will not attend for any further study visits. Participants without CKD (controls) will attend for a single study visit.

<u>End of the Trial</u>
The end of the study will be 15 years after the first study visit of the last recruited participant.

SELECTION AND WITHDRAWAL OF PARTICIPANTS

<u>Recruitment</u>
Participants with CKD will be recruited from nephrology clinics at multiple (planned to start with 11) participating centres.
The initial approach will be from a member of the patient's usual care team (which may include an investigator) either in person or by letter, and information about the trial will be on display in the relevant clinical areas. People who express an interest will be given a copy of the Participant Information Sheet and invited to contact the research team by telephone if they wish to participate or will have the opportunity to discuss the study at their next clinic visit. Potential participants who do not respond will be contacted once by phone, by a member of the usual care team, to check if they wish to participate.

Control participants (without CKD) will be recruited from among hospital staff members, people attending diabetes clinics and the general public via advertisements placed in the hospitals and in the press. People interested in participating will be invited to call a telephone number and will be sent a Participant Information Sheet by post. If they remain willing to participate, they will be invited to call back to arrange a study visit.

This protocol is confidential and the property of the University of Nottingham. No part of it may be transmitted, reproduced, published, or used by other persons without prior written authorisation from the University of Nottingham

The investigator or their nominee, e.g. from the research team or a member of the participant's usual care team, will inform the participant of all aspects pertaining to participation in the study. If needed, the usual hospital interpreter and translator services will be available to assist with discussion of the trial, the participant information sheets, and consent forms, but the consent forms and information sheets will not be available in other languages, other than Welsh.

The voluntary nature of the study will be explained to the potential participant who will also be reassured that clinical care will not be affected by their decision. The right of the participant to withdraw at any time will be explained. The participant's right not to attend follow-up visits will be explained as will the withdrawal of consent process. In the event of their withdrawal we will seek consent to use the data collected prior to withdrawal for analysis where appropriate. In the unlikely event that a participant withdraws consent for previously collected data to be used for analysis it will not be used. Similarly if a participant wishes previously stored samples not to be used, these will be destroyed.

Eligibility criteria: Participants with CKD

Inclusion criteria
1. Age Over 18 years of age
2. Estimated GFR 59-15ml/min/1.73m² or eGFR >60ml/min/1.73m² and urine albumin to creatinine ratio >30mg/mmol
3. Seen at least once in a Nephrology Clinic
4. Willing to participate in two study visits
5. Able to give informed consent and participate in study procedures

Exclusion criteria
1. Recipient of a solid organ transplant
2. Expected survival less than 1 year (in the opinion of local investigators)
3. Acute kidney injury within 3 months of recruitment
4. Major Acute Cardiovascular Event (MACE) within 3 months of recruitment
5. Primary Focal and Segmental Glomerulosclerosis and Idiopathic Minimal Change Disease
6. Current or planned chemotherapy for cancer

Eligibility criteria: Participants without CKD (controls)

Inclusion criteria
1. Age over 18 years of age
2. Willing to participate in one study visit
3. Able to give informed consent and participate in study procedures
4. No evidence of CKD
5. No other major illnesses except for hypertension and diabetes mellitus (50 participants will have diabetes but no CKD). Those with diabetes may have retinopathy or neuropathy.

Exclusion criteria

1. Estimated GFR<60ml/min/1.73m^2 in previous 12 months or last available result
2. Urine albumin to creatinine ratio >3mg/mmol in previous 12 months or last available result
3. Previous unilateral nephrectomy or partial nephrectomy

This protocol is confidential and the property of the University of Nottingham. No part of it may be transmitted, reproduced, published, or used by other persons without prior written authorisation from the University of Nottingham

4. Recipient of a solid organ transplant
5. Expected survival less than 1 year (in the opinion of local investigators)
6. Current or planned chemotherapy for cancer

Expected duration of participant participation
Study participants with CKD will participate in the study for 12-66 months of active follow-up. After that participants will be sent a questionnaire annually by post to assess quality of life (EQ-5D-5L) and health care resource utilisation during the previous year and we will collect outcome data without the need for further study visits; this annual follow up by questionnaire will continue for up to 14 years following their second study visit. Study participants acting as normal controls will attend only a single study visit.

Removal of participants from therapy or assessments/Participant Withdrawal
This is an observational study. Participants will be withdrawn from the study only if they withdraw their consent.

Informed consent
All participants will provide written informed consent. The Informed Consent Form will be signed and dated by the participant before they enter the trial. The Investigator will explain the details of the trial and provide a Participant Information Sheet, ensuring that the participant has sufficient time to consider participating or not. The Investigator will answer any questions that the participant has concerning study participation.

Informed consent will be collected from each participant before they undergo any interventions (including physical examination and history taking) related to the study. One copy of this will be kept by the participant, one will be kept by the Investigator, and a third will be retained in the patient's hospital records (paper or electronic).

Should there be any subsequent amendment to the final protocol, which might affect a participant's participation in the trial, continuing consent will be obtained using an amended Consent Form which will be signed by the participant. Should a participant be unable to attend the follow-up visit, they asked to sign the revised consent form having read the revised PIS. An opportunity to ask questions will be offered prior to signing. The signed consent form will be returned in a pre-paid envelope and then signed by the delegated member of the research team.

STUDY REGIMEN

Participants with CKD: Study visit 1:

After providing written informed consent participants will undergo the following assessments and study procedures:

Medical History: The following data will be collected by interview, questionnaire and examination of the participants' medical records.

- Socio-demographic: age, gender, ethnicity, first language, education status, marital status, employment, indices of multiple deprivation (IMD) score (derived from postcode of residence), smoking history, alcohol intake, dietary status (vegetarian/vegan)
- Medical: All previous illnesses with particular focus on CKD diagnosis, previous AKI, hypertension, diabetes, cardiovascular disease (CVD), atrial fibrillation, rheumatoid arthritis, thyroid disease, retinopathy (if diabetic), peripheral neuropathy (if diabetic). These will be used to calculate the Charleston Comorbidity Index.
- Family medical history: CVD before age 60 years, diabetes, CKD, ESKD.

This protocol is confidential and the property of the University of Nottingham. No part of it may be transmitted, reproduced, published, or used by other persons without prior written authorisation from the University of Nottingham

- All current medication including over the counter preparations and supplements/herbal preparations.
- Vaccination status for influenza and pneumococcus
- Prior laboratory results, especially prior serum creatinine results to enable analysis of trend in eGFR.
- The above data will be recorded on a questionnaire that participants will be asked to complete at or prior to the baseline study visit. Questionnaires will be checked by research staff at the study visit and participants will be assisted in completing the questionnaires if required. Details of the medical history and blood results will be verified by inspection of participants' hospital medical records.

Quality of Life and Functional Status

- Quality of life will be measured by EQ-5D-5L quality of life questionnaire
- Functional status will assessed using the Karnofsky score
- Current symptoms will be assessed using an Palliative Outcome Scale (POS) questionnaire (POS-Renal P7)
- Health literacy will be assessed by questionnaire (single question SILS)
- Cognitive function will be assessed with the "six item Cognitive Impairment Test" (6CIT) questionnaire
- Anxiety and depression symptoms will be assessed with the "Hospital Anxiety and Depression Scale" (HADS)

Anthropomorphic assessment:

- Height
- Weight
- Body Mass Index
- Hip to waist ratio
- Waist circumference
- Mid-upper arm muscle circumference
- Hand grip strength
- Blood pressure (seated; average of 3 readings recorded with an oscillometric device in accordance with British Hypertension Society guidelines)
- Urine dipstick test
- "Timed up and go" (TUG) test

Laboratory Assays:

The following tests to be performed as part of routine clinical care:

- Urea and electrolytes
- Estimated GFR
- Magnesium, calcium and phosphate
- Serum albumin
- Lipid profile (including triglycerides)
- Random blood glucose
- Haemoglobin A1C (if diabetic)
- Bicarbonate
- Uric acid
- Full blood count
- High sensitivity C-reactive protein (CRP)
- Ferritin (if anaemic)
- Folic acid (if anaemic)
- Vitamin B12 (if anaemic)

This protocol is confidential and the property of the University of Nottingham. No part of it may be transmitted, reproduced, published, or used by other persons without prior written authorisation from the University of Nottingham

- Serum Parathyroid hormone
- Urine albumin to creatinine ratio
- Urine protein to creatinine ratio

Specimens for Biorepository: In addition to the routine biochemistry detailed above, additional biosamples will be obtained from each participant at each study visit as follows:

- 10ml plasma (30mL of blood)
- 10ml serum (30mL of blood)
- 2 x 3ml whole blood for DNA extraction: genomics and epigenetics
- 1 x 2,5ml whole blood for RNA extraction
- 100ml urine

Core Biomarkers: A core set of 30 plasma and urine biomarkers that have previously been shown to have prognostic value in CKD will be measured in a central laboratory at University Hospital Geneva and the University of Geneva. A provisional list of these biomarkers is as follows: serum/plasma: fibroblast growth factor 23 (FGF23), NT Pro Brain Natriuretic Protein (NT Pro-BNP), high sensitivity troponin T, asymmetrical dimethyl arginine (ADMA) and symmetrical dimethyl arginine (SDMA); Adiponectin, Adrenomedullin, Vitamin D, Pentraxin 2, Galectin 3, neutrophil gelatinase-associated lipocalin (NGAL), cystatin C, interleukin(IL)6, IL-17, high sensitivity C reactive protein (hsCRP); transforming growth factor beta (TGF-β), tumour necrosis factor alpha (TNF-α), tumour necrosis factor-like weak inducer of apoptosis (TWEAK) urinary: transglutaminase 2 (uTG2), epidermal growth factor (uEGF), tissue inhibitor of matrix metalloproteinase 1 (uTIMP1), collagen 1 (uCol1), neutrophil gelatinase-associated lipocalin (uNGAL), kidney injury molecule-1(uKIM-1), Clusterin (uClusterin), Cadherin K (uCadherin K), matrix metalloproteinase 9 (uMMP-9), fibroblastspecific protein-1 (uFSP-1), N-acetyl-β-D-glucosaminidase (uNAG). However, it is anticipated that the list will change as new data become available during the recruitment phase of the study. Plasma, serum, whole blood and urine specimens will be stored until analysis in a central biorepository at the NIHR National Biosample Centre in Milton Keynes. Consent will be sought from participants for long term storage of biosamples and use of these biosamples in future studies.

Kidney biopsy tissue: Participants who have undergone a kidney biopsy for clinical diagnosis will be invited to participate in a sub-study that will comprise at least 15% of the whole cohort. In addition to enrolling in the study, these participants will be asked to consent for any surplus biopsy material, over and above that required for routine diagnostic purposes at the enrolling centre, to be utilised for the study. This will permit direct assessment of the disease processes that are causing kidney disease, to identify those factors that are present that are predictive of future decline in kidney function (including ESKD), and provide information that can subsequently be used to direct the development and evaluation of treatments.

Biopsy tissue that has been used in clinical laboratories for routine diagnosis will be transported to the Human Tissue Authority (HTA) licensed biorepository facility at University of Birmingham (Human Biomaterials Resource Centre - HBRC) for digital scanning and further analysis. This will include kidney biopsy sections analysed by clinical laboratories by staining including H&E, PAS and Masson's Trichrome. Clinical laboratories will also be asked to supply digital images of immunostaining and electron microscopy performed for clinical diagnosis if these are available.

Residual surplus kidney biopsy tissue will also be transported to the biorepository facility. From this surplus kidney biopsy tissue sections will be cut for additional assessment of the pathological changes associated with CKD by specialised stains and immunostaining.

This protocol is confidential and the property of the University of Nottingham. No part of it may be transmitted, reproduced, published, or used by other persons without prior written authorisation from the University of Nottingham

Digital images will be analysed to provide:
- Confirmation and further classification of clinical diagnosis
- Automated assessment of glomerulosclerosis, extent of interstitial fibrosis, inflammation, peritubular capillary density, collagen accumulation, resident and infiltrating cell phenotype

Tissue sections will also be used for RNA extraction and analysis of gene expression. Surplus tissue will be stored in the University of Birmingham Biorepository facility and sections will be supplied to investigators as required. Consent will be sought from participants for long-term storage of residual kidney biopsy tissue and use of this tissue in future studies.

Participants acting as controls: Study Visit 1:

After providing written informed consent participants will undergo the following assessments and study procedures:

Socio-Demographic data:
Participants will be asked to give their sex and date of birth as well as post code of residence (to derive indices of multiple deprivation score)

Anthropomorphic Assessment:
- Height
- Weight
- Body Mass Index
- Hip to waist ratio
- Waist circumference

Laboratory Assays:
- Urea and electrolytes
- Estimated GFR
- Urine albumin to creatinine ratio
- Random blood glucose
- High sensitivity C-reactive protein
- Haemoglobin A1C (if diabetic)

Specimens for Biorepository: In addition to the biochemistry detailed above, additional biosamples will be obtained as follows:
- 10ml plasma (30mL of blood)
- 10ml serum (30mL of blood)
- 100ml urine
- 2 x 3ml whole blood for DNA extraction: genomics and epigenetics
- 1 x 2,5ml whole blood for RNA extraction

Participants with CKD: Study Visit 2

Participants will be asked to attend a second study visit 12-66 months from the date of the first study visit. The assessments and procedures for the second study visit will be the same as for the first study visit. In addition, participants will be asked to complete a health utilisation

This protocol is confidential and the property of the University of Nottingham. No part of it may be transmitted, reproduced, published, or used by other persons without prior written authorisation from the University of Nottingham

questionnaire to obtain details regarding hospital admissions, GP visits and medication changes during the year since recruitment

Compliance

Compliance will be defined by attendance at the second study visit.

Criteria for terminating trial

The study will be discontinued only if for unforeseen circumstances it becomes clear that it is no longer feasible. If a participating centre is unable to recruit successfully, it will be withdrawn from the study and replaced by another centre. Participants already recruited from a centre that withdraws from the study will remain in the study.

RADIATION EXPOSURE

Not applicable

TRANSPORT AND STORAGE OF THE TISSUES

Samples will be stored in a linked anonymised format and labelled using a barcode linked to the study number to permit accurate linkage to study data and the consent form.

Samples for NHS pathology analysis will be labelled in accordance with local NHS procedures.

Serum, plasma and urine samples will be stored at each participating site initially at -20 degrees centigrade and transferred to -80 degrees centigrade within 72 hours of collection.
Frozen samples will be transferred on dry ice by courier from each participating site to the NIHR National Biosample Centre in Milton Keynes approximately every 4 months. All shipments will contain a complete inventory of all samples, along with the name of the person responsible for sending the samples. After completion of recruitment and follow-up, samples will similarly shipped from the National Biosample Centre to participating laboratories for analysis. Any sample left after analysis will either be returned to the National Biosample Centre or destroyed. A master database of all frozen samples will be held at the National Biosample Centre in a password protected file.

Surplus kidney biopsy tissue embedded in paraffin blocks as well as stained microscope slides will be shipped at room temperature from each participating centre to the University of Birmingham Biorepository Centre. A small number of frozen surplus kidney biopsy specimens will be shipped on dry ice. At the University of Birmingham Biorepository Centre slides will be photographed and digital images will be stored and identified using the participant identification number (PIN). The slides will then be returned by courier to the participating centre. Residual renal tissue in paraffin blocks will be further sectioned and placed on microscope slides for further analyses as planned by the investigators. These slides will be labelled with the PIN. Slides will be shipped to participating laboratories for analysis. A master database of all biopsy samples will be held at the University of Birmingham Biorepository Centre in a password protected file. Tissues not used by the end of the study will be returned to the Pathology Department of origin. If the surplus tissue is required by the clinical centre for further routine testing at any time, then it will be returned from the biorepository.

LABORATORY ANALYSES

Routine biochemistry tests will be performed in local NHS clinical biochemistry laboratories. Novel biomarkers in serum, plasma and urine will be assayed in research laboratories. DNA

This protocol is confidential and the property of the University of Nottingham. No part of it may be transmitted, reproduced, published, or used by other persons without prior written authorisation from the University of Nottingham

will be extracted from whole blood samples at the NIHR National Biosample Centre and stored for subsequent analysis.

OUTCOMES

Data regarding the primary and secondary outcomes will be obtained from multiple sources as summarised below:

**Outcome**	**Source**
ESKD	UK Renal Registry; and local laboratory results via Patient View
CKD Progression	Local laboratory results via Patient View
Death	Office of National Statistics (ONS) through Digital Health and Care Wales (DHCW)
Hospital admission	Hospital Episode Statistics (HES) through DHCW
AKI	HES through DCHW
Infections	HES through DHCW
MACE	ONS and HES through DHCW
Fractures	HES through DHCW
New Cancers	ONS Cancer Registry through DHCW

An adjudication committee will be established at each site to verify and adjudicate outcome events including cause of death, cardiovascular events and others as required by evaluation of individual medical records.

STATISTICS

<u>Methods</u>

Standard descriptive statistics will be used to describe patients at baseline (sociodemographic and clinical characteristics, laboratory parameters and biomarkers, quality of life). Summary statistics (mean, SD, 95% confidence intervals, median IQR), frequency (percent) distribution tests and parametric (t-test) and non-parametric statistical methods will be used to assess group differences, as appropriate. Normality of the distributions will be assessed by Kolmogorov-Smirnov or Shapiro-Wilk tests, as appropriate, with logtransformation, as appropriate. Adjustment for multiple comparisons will be made by posthoc tests (e.g., Tukey), as necessary.

Analyses of changes over time of selected clinical characteristics and biomarkers will be performed. Changes in serum creatinine levels and eGFR will be analysed using mixedeffects models and generalized estimation equations. The latter approaches facilitate handling of repeated measurements and the heterogeneity of individual creatinine levels.

Time-to-event analyses (Kaplan-Meier, Cox regression) will be the principal approach to analysing end points such as all-cause mortality, cardiovascular and ESKD providing the proportionality assumption is met. A competing risk framework will be used. Also, logistic regression methods will be used for dichotomous outcomes in a fixed time period (e.g., progression of CKD) with time-independent covariates and Poisson regression will be used for count data (e.g., number of hospitalisations). Multivariable regression models will be used to study the associations between patient characteristics, biomarkers and the various study outcomes.

This protocol is confidential and the property of the University of Nottingham. No part of it may be transmitted, reproduced, published, or used by other persons without prior written authorisation from the University of Nottingham

Sample size and justification

The study aims to recruit 3000 patients from 11 centres. This is a pragmatic approach based on feasibility and cost, and is similar to other large well phenotyped CKD cohorts (e,g. CRIC 4000 [8], CKD Rein 3600 [9], German CKD 5000 [10]. It is not driven by a sample size based on a hypothesis for a specific primary outcome, however, a post-hoc estimation of power for each of the main comparisons and relationships will be performed. 100 participants without CKD (50 with type 2 diabetes mellitus and 50 without diabetes) will be recruited to act as controls for the biomarker assays.

The main analyses will be multivariable looking at the predictive performance of biomarkers alone and in combination with other socio- demographic and clinical variables. A rule of thumb is that 10 events of interest are required per predictor (variable) in a final multivariable prognostic model. It is assumed also that the event rates will be dependent on a variety of factors in the recruited cohort such as on age, eGFR, co-morbidity and ethnic status, so they cannot be accurately specified a priori. However, initial estimates from the UK CRISIS study, which included participants similar to those required for NURTuRE-CKD indicated event rates of 5/100 person years for death (mortality) and 7-10/100 person years for ESKD [11].

Therefore, the planned initial 5.5-year follow up of a 3000 person cohort should generate >100 events for all the key outcomes (deaths, ESKD, CV events, AKI, CKD progression). Such an a priori approximate estimation of study sample size and power, for logistic reasons only, may be based on the consideration for a minimum hazard ratio (HR) that could be detected for a baseline biomarker in time-to-event survival analysis (e.g. Kaplan-Meier) for an outcome such as mortality. In this sense, two approaches are available: balanced and unbalanced (unequal-size) groups.

For example, if the baseline frequency of a biomarker level is 50% (i.e., a total of 3000 patients or 2 equal groups of 1500 patients each) then a minimum HR of 1.52-1.70 for death (i.e. mortality of minimum 5% in the second, control group) can be detected with 80% power at two-sided 5% significance level (required minimum number of 114-182 events). For a less common biomarker, with a prevalence of 10% (n=300 patients), the minimum HR would rise to 2.34.

Similarly for ESKD as an outcome, if the baseline frequency of a biomarker level is 50% (i.e., a total of 3000 patients or 2 equal groups of 1500 patients each) then a minimum HR of 1.391.47 for ESKD (i.e., event rate of minimum 8.5% in the second, control group) can be detected with 80% power at two-sided 5% significance level (required minimum number of 210-296 events). For a less common biomarker, with a prevalence of 10% (n=300 patients), the minimum HR would rise to 1.98.

Multiple imputation will be used to deal with missing data.

Definition of populations analysed

All participants who complete the baseline study visit will be included in the primary analysis. All participants who complete the baseline and follow-up study visits will be included in analyses of change in baseline variables over time as well as the relationship of these changes to subsequent outcome events.

Subgroup analysis will be conducted as indicated by the research question being investigated. Pre-specified subgroups will include:

- Participants 65 years and older versus younger than 65 years
- Male versus female participants
- White versus Black versus South Asian participants
- Participants with diabetes mellitus versus without diabetes

This protocol is confidential and the property of the University of Nottingham. No part of it may be transmitted, reproduced, published, or used by other persons without prior written authorisation from the University of Nottingham

- Participants with pre-existing cardiovascular disease versus without CVD
- CKD categories (as specified by the KDIGO and NICE Classification)

Health Economics Analyses

Health economics analysis will form part of the study. Costs will be taken from the NHS and PSS perspective. A questionnaire will collect health resource use information from participants including medications, primary care visits, outpatient visits, A&E attendances and hospital admissions. These data will be combined with HES data and data from Renal Department databases to assess total health resource utilisation. Health related quality of life will be measured annually by EQ-5D-5L.

The UK tariff will be used to translate the EQ-5D-5L questionnaire into utility scores. Area under the curve methods will be used to calculate the quality adjusted life year (QALY). Generalised linear mixed models will be employed to estimate the costs and QALYs associated with baseline characteristics including socio-economic groups, and clinical factors.

ADVERSE EVENTS

The primary and secondary endpoints of this study are all adverse events and data regarding these AEs will be collected and recorded as described in the protocol. As this is an observational study, the only AEs with a causal relationship to study procedures will be those directly related to study visits, which are expected to be extremely uncommon. Only SAEs and SUSARs related to study visits will be reported. Participants will be asked to report only AEs that could be related to study visits.

ETHICAL AND REGULATORY ASPECTS

ETHICS COMMITTEE AND REGULATORY APPROVALS

The trial will not be initiated before the protocol, informed consent forms and participant and GP information sheets have received approval / favourable opinion from the Research Ethics Committee (REC), and the respective National Health Service (NHS) Research & Development (R&D) department. Should a protocol amendment be made that requires REC approval, the changes in the protocol will not be instituted until the amendment and revised informed consent forms and participant information sheets (if appropriate) have been reviewed and received approval / favourable opinion from the REC and R&D departments. A protocol amendment intended to eliminate an apparent immediate hazard to participants may be implemented immediately providing that the REC are notified as soon as possible and an approval is requested. Minor protocol amendments only for logistical or administrative changes may be implemented immediately; and the REC will be informed.

The trial will be conducted in accordance with the ethical principles that have their origin in the Declaration of Helsinki, 1996; the principles of Good Clinical Practice, and the Department of Health Research Governance Framework for Health and Social care, 2005.

INFORMED CONSENT AND PARTICIPANT INFORMATION

The process for obtaining participant informed consent will be in accordance with the REC guidance, and Good Clinical Practice (GCP) and any other regulatory requirements that might be introduced. The investigator or their nominee and the participant shall both sign and date the Informed Consent Form before the person can participate in the study.

This protocol is confidential and the property of the University of Nottingham. No part of it may be transmitted, reproduced, published, or used by other persons without prior written authorisation from the University of Nottingham

The participant will receive a copy of the signed and dated forms and the original will be retained in the Trial Master File. A second copy will be filed in the participant's medical notes and a signed and dated note made in the notes (paper or electronic) that informed consent was obtained for the trial.

The decision regarding participation in the study is entirely voluntary. The investigator or their nominee shall emphasize to them that consent regarding study participation may be withdrawn at any time without penalty or affecting the quality or quantity of their future medical care, or loss of benefits to which the participant is otherwise entitled. No trial-specific interventions will be done before informed consent has been obtained.

The investigator will inform the participant of any relevant information that becomes available during the course of the study, and will discuss with them, whether they wish to continue with the study. If applicable they will be asked to sign revised consent forms.

If the Informed Consent Form is amended during the study, the investigator shall follow all applicable regulatory requirements pertaining to approval of the amended Informed Consent Form by the REC and use of the amended form (including for ongoing participants).

RECORDS

Case Report Forms

Each participant will be assigned a participant identification number, allocated at recruitment, for use on CRFs, other trial documents and any electronic database or spreadsheet used for analysis. The documents and database will also use their initials (of first and last names separated by a hyphen) and year of birth.

This study will use electronic CRFs stored on a secure server at the UK Renal Registry (UKRR). The UKRR database will include participant identifiable data including names, date of birth, address, NHS and hospital numbers to facilitate collection of outcome data and routine biochemical data. The UKRR routinely deals with confidential patient data and has robust systems in place to ensure security and confidentiality of these data. CRFs will be treated as confidential documents and held securely in accordance with regulations. Access to CRFs shall be restricted to those personnel approved by the Chief or local Principal Investigator and recorded on the 'Trial Delegation Log.' Data that are exported from the UKRR database will have all participant identifiable data removed.

The investigator will make a separate confidential record of the participant's name, date of birth, local hospital number or NHS number, and Participant Trial Number (the Trial Recruitment Log), to permit identification of all participants enrolled in the trial, in accordance with regulatory requirements and for follow-up as required. This record will be held on a secure server in a password protected file.

Sample Labelling

Each participant will be assigned a participant trial identity code number (PIN) for use on the consent forms and other study documents and the electronic database. Samples will be labelled with a barcode linked to the PIN. The documents and database will also use their initials (of first and last names separated by a hyphen) and year of birth

Samples for NHS pathology analysis will be labelled in accordance with local NHS procedures. Samples for storage and later analysis in research laboratories will be labelled a barcode linked to the PIN.

This protocol is confidential and the property of the University of Nottingham. No part of it may be transmitted, reproduced, published, or used by other persons without prior written authorisation from the University of Nottingham

Source documents

Source documents will be filed at the investigator's site and may include but are not limited to, consent forms, current medical records, laboratory results and records. A CRF may also completely serve as its own source data. Only trial staff as listed on the Delegation Log shall have access to trial documentation other than the regulatory requirements listed below.

Direct access to source data / documents

The CRF and all source documents, including progress notes and copies of laboratory and medical test results shall made be available at all times for review by the Chief Investigator, Sponsor's designee and inspection by relevant regulatory authorities (e.g. DH, Human Tissue Authority).

DATA PROTECTION

All trial staff and investigators will endeavour to protect the rights of the trial's participants to privacy and informed consent, and will adhere to the Data Protection Act, 2018. The CRF will only collect the minimum required information for the purposes of the trial. CRFs will be held on a secure server with password-protected access by the UK Renal Registry. Access to the information will be limited to the trial staff and investigators and relevant regulatory authorities (see above). Computer held data including the trial database will be held securely and password protected. All data will be stored on a secure dedicated web server. Access will be restricted by user identifiers and passwords (encrypted using a one way encryption method). Information about the trial in the participant's medical records / hospital notes will be treated confidentially in the same way as all other confidential medical information.

Electronic data will be backed up every 24 hours to both local and remote media in encrypted format.

QUALITY ASSURANCE & AUDIT

INSURANCE AND INDEMNITY

Insurance and indemnity for trial participants and trial staff is covered within the NHS Indemnity Arrangements for clinical negligence claims in the NHS, issued under cover of HSG (96)48. There are no special compensation arrangements, but trial participants may have recourse through the NHS complaints procedures.

The University of Nottingham as research Sponsor indemnifies its staff, research participants and research protocols with both public liability insurance and clinical trials insurance. These policies include provision for indemnity in the event of a successful litigious claim for proven non-negligent harm.

TRIAL CONDUCT

Trial conduct may be subject to systems audit of the Trial Master File for inclusion of essential documents; permissions to conduct the trial; Trial Delegation Log; CVs of trial staff and training received; local document control procedures; consent procedures and recruitment logs; adherence to procedures defined in the protocol (e.g. inclusion / exclusion criteria, correct randomisation, timeliness of visits); adverse event recording and reporting; accountability of trial materials and equipment calibration logs.

This protocol is confidential and the property of the University of Nottingham. No part of it may be transmitted, reproduced, published, or used by other persons without prior written authorisation from the University of Nottingham

The study Project Manager or, where required, a nominated designee of the Sponsor, shall carry out a site systems audit at least yearly and an audit report shall be made to the Study Advisory Group.

TRIAL DATA

Monitoring of trial data shall include confirmation of informed consent; source data verification; data storage and data transfer procedures; local quality control checks and procedures, backup and disaster recovery of any local databases and validation of data manipulation. The study Project manager, or where required, a nominated designee of the Sponsor, shall carry out monitoring of trial data as an ongoing activity.

Entries on CRFs will be verified by inspection against the source data. A sample of CRFs (10% or as per the study risk assessment) will be checked during recruitment and follow-up phases for verification of all entries made. In addition, the subsequent capture of the data on the trial database will be checked. Where corrections are required, these will carry a full audit trail and justification.

Trial data and evidence of monitoring and systems audits will be made available for inspection by REC as required.

To facilitate data security during collaborative projects, a copy of all pseudonymised study data will be transferred from the UK Renal Registry to a "Trusted Research Environment" (TRE) hosted by the SAIL Databank, which is owned by Swansea University. Further, the UK Renal Registry will provide Digital Health and Care Wales (DHCW) with participant identifiers to enable DHCW to establish linkage of participant data to the HES and ONS databases, as facilitated by the partnership between NURTuRE and the British Heart Foundation Data Science Centre Cohorts Platform (based within the SAIL Databank). DHCW act as a linkage broker by sharing identifiers to any non-Welsh NHS bodies for linkage in those jurisdictions (England, Scotland, NI where relevant). Once the linkage has been established, participant identifiable data will be deleted by DHCW and the corresponding national health record providers in other UK constituent countries. Following this change, partners approved by the NURTuRE Strategy Committee will access the data via the TRE and will not receive physical copies of the data. Users will not be able to export the original data from the TRE. This arrangement will therefore facilitate improved data security.

The UK Renal Registry will continue to hold a copy of the pseudonymised data and participant identifiers but will act only as a Data Processor. The UK Renal Registry will send annual updates of pseudonymised data to the SAIL databank.

RECORD RETENTION AND ARCHIVING

In compliance with the ICH/GCP guidelines, regulations and in accordance with the University of Nottingham Research Code of Conduct and Research Ethics, the Chief or local Principal Investigator will maintain all records and documents regarding the conduct of the study. These will be retained for at least 7 years or for longer if required. If the responsible investigator is no longer able to maintain the study records, a second person will be nominated to take over this responsibility.

The Trial Master File and trial documents held by the Chief Investigator on behalf of the Sponsor shall be finally archived at secure archive facilities at the University of Nottingham. This archive shall include all trial databases and associated meta-data encryption codes.

This protocol is confidential and the property of the University of Nottingham. No part of it may be transmitted, reproduced, published, or used by other persons without prior written authorisation from the University of Nottingham

DISCONTINUATION OF THE TRIAL BY THE SPONSOR

The Sponsor reserves the right to discontinue this trial at any time for failure to meet expected enrolment goals, for safety or any other administrative reasons. The Sponsor shall take advice from the Trial Steering Committee and Data Monitoring Committee as appropriate in making this decision.

STATEMENT OF CONFIDENTIALITY

Individual participant medical information obtained as a result of this study is considered confidential and disclosure to third parties is prohibited with the exceptions noted above. Participant confidentiality will be further ensured by utilising PIN code numbers to correspond to treatment data in the computer files.

Medical information may be given to the participant's medical team and all appropriate medical personnel responsible for the participant's welfare. This will be made clear in the patient information sheet and during the consent process.

If information is disclosed during the study that could pose a risk of harm to the participant or others, the researcher will discuss this with the CI and where appropriate report accordingly.

Data generated as a result of this trial will be available for inspection on request by the participating physicians, the University of Nottingham representatives, the REC, local R&D Departments and the regulatory authorities.

PUBLICATION AND DISSEMINATION POLICY

Study results will be presented at national and international scientific conferences and will be published in peer-reviewed journals. It is anticipated that the study will generate a large number of presentations and publications over several years, starting with publication of baseline data. Participants will not be identified in any presentations or publications.

USER AND PUBLIC INVOLVEMENT

This protocol has been developed with input from patient advisers and has incorporated feedback received from participants in four previous cohort studies of CKD conducted by the investigators in the UK. The Study Advisory Group will include a patient representative who will contribute to the oversight of the study and provide advice from a patient perspective as required.

STUDY FINANCES

<u>Funding source</u>

This study is funded by a grant from Kidney Research UK supported by funding from several pharmaceutical and biodiagnostic companies. The companies have been active collaborators in the development of the protocol and at time of submission comprise:

UCB Pharma Ltd
AbbVie
Evotec International GmbH
AstraZeneca
Retrophin Inc.

This protocol is confidential and the property of the University of Nottingham. No part of it may be transmitted, reproduced, published, or used by other persons without prior written authorisation from the University of Nottingham

Participant stipends and payments

Participants will not be paid to participate in the trial. Travel expenses will be offered for any hospital visits in excess of usual care.

This protocol is confidential and the property of the University of Nottingham. No part of it may be transmitted, reproduced, published, or used by other persons without prior written authorisation from the University of Nottingham

SIGNATURE PAGES

Signatories to Protocol:

Chief Investigator: Prof M.W.Taal

Signature:

Date:

Co- investigator: Prof D. Wheeler

Signature:______ ______________________________

Date: ___________

Co- investigator: Dr S. Fraser

Signature:_____ _______________________________

Date:

Co- investigator: Prof P. Cockwell

Signature:____________________________________

Date: ___________

Co- investigator: Prof P. Kalra

Signature:____________________________________

Date: ___________

Trial Statistician: Dr R. Steenkamp

This protocol is confidential and the property of the University of Nottingham. No part of it may be transmitted, reproduced, published, or used by other persons without prior written authorisation from the University of Nottingham

Signature:___________________________________

Date: ___________

This protocol is confidential and the property of the University of Nottingham. No part of it may be transmitted, reproduced, published, or used by other persons without prior written authorisation from the University of Nottingham

REFERENCES

1. Jha V, Garcia-Garcia G, Iseki K*, et al.* Chronic kidney disease: global dimension and perspectives. Lancet. 2013 Jul 20;382(9888):260-72.
2. Astor BC, Matsushita K, Gansevoort RT*, et al.* Lower estimated glomerular filtration rate and higher albuminuria are associated with mortality and end-stage renal disease. A collaborative meta-analysis of kidney disease population cohorts. Kidney Int. 2011 Feb 2;79:1331-40.
3. Gansevoort RT, Matsushita K, van der Velde M*, et al.* Lower estimated GFR and higher albuminuria are associated with adverse kidney outcomes in both general and highrisk populations. A collaborative meta-analysis of general and high-risk population cohorts.
Kidney Int. 2011 Feb 2;80:93-104.
4. Matsushita K, van der Velde M, Astor BC*, et al.* Association of estimated glomerular filtration rate and albuminuria with all-cause and cardiovascular mortality in general population cohorts: a collaborative meta-analysis. Lancet. 2010 Jun 12;375(9731):2073-81.
5. Tangri N, Grams ME, Levey AS*, et al.* Multinational Assessment of Accuracy of Equations for Predicting Risk of Kidney Failure: A Meta-analysis. JAMA. 2016 Jan 12;315(2):164-74.
6. Tangri N, Stevens LA, Griffith J*, et al.* A predictive model for progression of chronic kidney disease to kidney failure. JAMA. 2011 Apr 20;305(15):1553-9.
7. Schievink B, Lambers Heerspink H, Leufkens H*, et al.* The use of surrogate endpoints in regulating medicines for cardio-renal disease: opinions of stakeholders. PLoS One. 2014;9(9):e108722.
8. Denker M, Boyle S, Anderson AH*, et al.* Chronic Renal Insufficiency Cohort Study (CRIC): Overview and Summary of Selected Findings. Clin J Am Soc Nephrol. 2015 Nov 6;10(11):2073-83.
9. Stengel B, Combe C, Jacquelinet C*, et al.* The French Chronic Kidney Disease-Renal Epidemiology and Information Network (CKD-REIN) cohort study. Nephrol Dial Transplant. 2014 Aug;29(8):1500-7.
10. Titze S, Schmid M, Kottgen A*, et al.* Disease burden and risk profile in referred patients with moderate chronic kidney disease: composition of the German Chronic Kidney Disease (GCKD) cohort. Nephrol Dial Transplant. 2015 Mar;30(3):441-51.
11. Hoefield RA, Kalra PA, Baker P*, et al.* Factors associated with kidney disease progression and mortality in a referred CKD population. Am J Kidney Dis. 2010 Dec;56(6):1072-81.

This protocol is confidential and the property of the University of Nottingham. No part of it may be transmitted, reproduced, published, or used by other persons without prior written authorisation from the University of Nottingham
